# Epitaxial Stabilization and Persistent Nucleation of the 3C Polymorph of Ba_0.6_Sr_0.4_MnO_3_

**DOI:** 10.1021/acsami.3c11934

**Published:** 2024-01-17

**Authors:** Catherine Zhou, Charles Evans, Elizabeth C. Dickey, Gregory S. Rohrer, Paul A. Salvador

**Affiliations:** Department of Materials Science and Engineering, Carnegie Mellon University, 5000 Forbes Avenue, Pittsburgh, Pennsylvania 15213, United States

**Keywords:** interval pulsed laser deposition, epitaxial thin films, synthesis, metastable, polytypes, perovskites, oxides

## Abstract

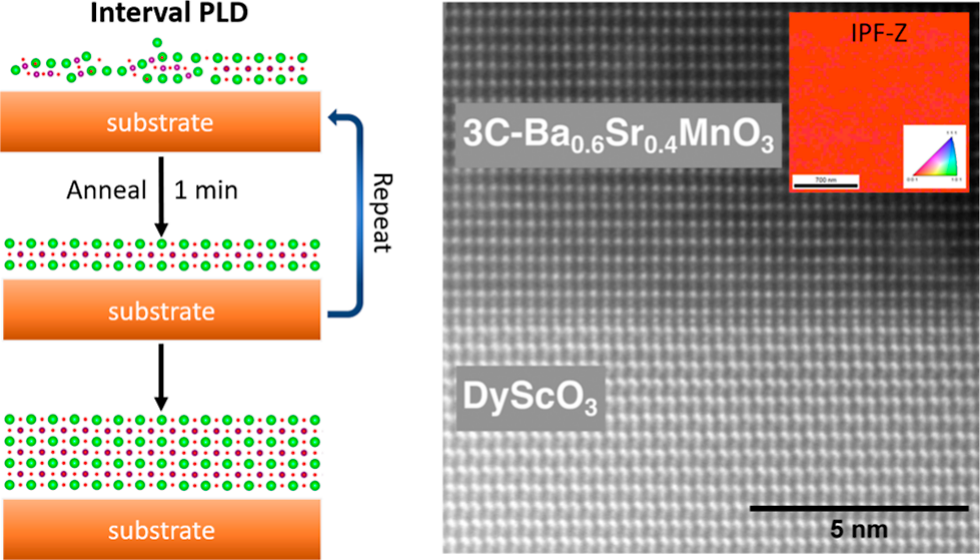

Ba-rich compositions in the Ba_*x*_Sr_1–*x*_MnO_3_ (BSMO) cubic perovskite (3C) system are magnetic ferroelectrics and are of interest for their strong magnetoelectric coupling. Beyond *x* = 0.5, they only form in hexagonal polymorphs. Here, the 3C phase boundary is pushed to Ba_0.6_Sr_0.4_MnO_3_ for the thin films. Using regular pulsed laser deposition (rPLD), 3C Ba_0.6_Sr_0.4_MnO_3_ could be epitaxially stabilized on DyScO_3_ (101)_o_ substrates by using a 0.1% O_2_/99.9% N_2_ gas mixture. However, the 3C phase was mixed with the 4H polymorph for films 24 nm thick and above, and the films were relatively rough. To improve flatness and phase purity, changes in growth kinetics were investigated and interval PLD (iPLD) was especially effective. In iPLD, deposition is interrupted after completion of approximately one monolayer, and the deposit is annealed for a specific period of time before repeating. Both film flatness and, more importantly, the volume of the 3C polymorph improved with iPLD, resulting in 40 nm single-phase films. The results imply that iPLD improves the persistent nucleation of highly metastable phases and offers a new approach to epitaxial stabilization of novel materials, including more Ba-rich BSMO compositions in the 3C structure.

## 1 Introduction

Cubic alkaline earth manganite perovskites have been the focus of many computational and physical studies because of their potential multiferroic properties, in which the ferromagnetic and ferroelectric orders originate from the electrons on the Mn^4+^ ions and their bonding with oxygen, resulting in a potentially strong magnetoelectric coupling around 200 K.^1−8^ Increasing the size of the cubic perovskite unit cell has been predicted to enhance the noncentrosymmetric distortion (off-centering of the Mn within the octahedra) and ferroelectric instability in the manganite system.^3,4,9−11^ For metastable cubic SrMnO_3_, strain engineering or chemical expansion via substitution with Ba has been used to expand the unit cell and induce a ferroelectric phase.^5,7^ Cubic BaMnO_3_ has never been synthesized experimentally, but has been predicted to be antiferromagnetic and ferroelectric without strain, with an electric polarization of 12 μC/cm^2^.^4^ Furthermore, a ferromagnetic phase is computationally predicted under tensile strain.^3,11^ While the novel multiferroic properties of BaMnO_3_ are enticing, the cubic perovskite is the metastable phase. In the Ba_*x*_Sr_1–*x*_MnO_3_ (BSMO) system, hexagonal perovskite polytypes are the stable structures, and increasing the Ba concentration, *x*, continually destabilizes the metastable cubic structure.^12−14^ It therefore continues to be of interest to find synthesis methods to stabilize the cubic structure in Ba-rich BSMO compounds.

The ground-state preferences as a function of tolerance factor,^15,16^ Sr or Ba compositions,^13^ and oxygen content are discussed in Supporting Information S1. The ground-state structures for BaMnO_3_ and SrMnO_3_ are described as two-layered (2H) and four-layered (4H) hexagonal structures,^13^ respectively, having all face-sharing MnO_6_ octahedra (2H) or alternating corner-sharing and face-sharing MnO_6_ octahedra (4H). The metastable cubic structure is a three-layered (3C) cubic structure with all corner-sharing octahedra.^13^ In bulk synthesis methods, high-temperature, low-oxygen pressure, or high-pressure conditions have been found to stabilize cubic stacking (corner-sharing octahedra) in compositions with *x* ≤ 0.5 in BSMO.^8,16−18^ Using solid-state methods, Goian et al.^17^ were able to synthesize single-phase 3C BSMO up to *x* = 0.45. The highest 3C BSMO composition, with *x* = 0.50, was first synthesized using single-crystal techniques by Sakai et al.^8^ Both of these studies relied on oxygen-deficient synthesis conditions followed by an oxidation annealing step to produce single-phase 3C BSMO. Furthermore, 3C BaMnO_3_ was predicted to be stabilized above 20 GPa,^11^ but a new polymorph, a 6H one, was found instead by Qin et al.^19^ It is clear that stabilizing a 3C BSMO compound will be increasingly difficult with higher Ba concentrations and that additional parameters other than temperature and pressure are needed to achieve the desired phase.

Epitaxial stabilization is one method that offers an additional structure-directing thermodynamic parameter (the interface with the substrate).^20−26^ BSMO phases have been epitaxially stabilized in the 3C polytype using regular pulsed laser deposition (rPLD), in synthesis conditions that would otherwise favor hexagonal polymorphs, but only for *x* ≤ 0.5.^14^ Following the work of Sakai et al.,^8^ Langenberg et al.^14^ studied the effect of strain, temperature, and oxygen partial pressure on the epitaxial stability of the 3C phase with the increasing Ba content, describing a feasible route to synthesize metastable phases with novel functional properties.^27^ They found that substrates that induce an in-plane tensile strain lead to better 3C stabilization, presumably due to the increase in volume and a higher Mn^3+^/Mn^4+^ ratio during growth.^14,28^ This hypothesis was later confirmed by the same group using differently strained SrMnO_3_ films on perovskite substrates.^28^ Consistent with bulk trends, higher substrate temperatures and lower oxygen pressures stabilize the 3C phase with increasing Ba content due to the increased concentration of oxygen vacancies and subsequent lattice expansion.^12,14,29−32^ Furthermore, they observed a maximum thickness under which single-phase 3C films could be grown, and this thickness decreases to less than 10 nm for BSMO *x* = 0.5.^14^ Above this maximum thickness, the 4H phase begins to nucleate and film peaks are more difficult to detect via X-ray diffraction (XRD) due to the lower structure factor of the 4H orientation that is stabilized on (100) substrates. Zhou et al.^26^ computed that 3C BaMnO_3_ may be epitaxially stabilized over 4H below 2 stoichiometric layers, indicating that further investigations on the epitaxial stabilization of BSMO with *x* > 0.5 may be fruitful.

The nucleation outcomes in epitaxial growth are controlled by both thermodynamic and the kinetic aspects of nucleation, which are themselves related to process variables. These include temperature (*T*) and oxygen partial pressure (*P*_O_2__), which clearly impact thermodynamic phase stability, as well as total pressure (*P*_t_), laser energy (*E*), and laser frequency (*f*), which control nucleation thermodynamics and kinetics. These parameters also impact the growth mode and film roughness (which can then influence nucleation). Interval pulsed laser deposition (iPLD herein) provides another kinetic parameter to impact growth: a rest period that allows for some relaxation of the prior deposit and has been used to promote layer-by-layer (LBL) growth in thin films.^33−37^ Koster et al.^33^ first introduced the iPLD method in 1999 for the homoepitaxial deposition of flat SrTiO_3_. In that work, a high laser frequency of 100 Hz was used to deposit one monolayer, and this was followed by an annealing period; this cycle was repeated until the desired film thickness was achieved. The high laser repetition rate increases supersaturation of the surface and limits adatom diffusion while also promoting interlayer mass transport. Subcritical clusters in each cycle become unstable with time, so the interval (or period of rest without deposition) allows these clusters to dissociate into mobile atoms and leads to smoother films.^37,38^ Motivated by the promise of iPLD to promote LBL growth, Chen et al.^37^ investigated the effect of lower laser frequencies (1–10 Hz), which are more common in existing PLD systems. Even depositing multiple layers prior to the rest interval (between 0.2 and 30 s), reconstruction of atomically smooth surfaces was still achieved, and LBL growth persisted.

There are no reports of improving nucleation outcomes of metastable phases using iPLD, which is a little surprising because one can expect that retention of the flat structure-directing interface throughout growth and reduction in defect accumulation improve the likelihood that the nucleation and retention of an epitaxially stabilized phase persist throughout growth. There are examples of the improved order of Cr and Re in Sr_2_CrReO_6_ double perovskite films^39^ and of layers in (Sr, Ca) CuO_2_–BaCuO_2_ superlattices^35^ using iPLD. The goal of this paper is twofold: to move the 3C phase boundary beyond *x* = 0.5 using epitaxial stabilization, specifically to BSMO *x* = 0.6, and to demonstrate that single-phase films can be stabilized well beyond the previously reported maximum thickness of 5–10 nm,^14^ specifically using iPLD. We first explore the phase stability of 3C BSMO (*x* = 0.6) films on DyScO_3_ (101)_o_ single-crystal substrates using rPLD in conditions modified slightly from prior work.^14^ We then explore deposition conditions in rPLD to address the problem of surface roughness at high substrate temperatures and low oxygen partial pressures, but these have negative consequences for 3C phase formation. Finally, we investigated whether iPLD can improve the formation of the 3C phase, successfully pushing the single-phase thickness to 40 nm. Overall, these observations indicate that even higher Ba-content materials should be realizable using persistent nucleation in iPLD.

## 2 Experimental Methods

4H Ba_0.6_Sr_0.4_MnO_3_ targets were fabricated using conventional ceramic synthesis methods (described in Supporting Information S2.1).^40,41^ 1 mm-thick single crystals (MTI Corporation) of orthorhombic (o) DyScO_3_ (DSO) having (101)_o_ orientations (which is equivalent to a pseudocubic (pc) (001)_pc_) were used as substrates for most depositions. The lattice parameters for o-DSO are *a* = 5.720 Å, *b* = 7.903 Å, and *c* = 5.442 Å. The orthogonal in-plane pseudocubic lattice parameters are  = 3.948 Å and  = 3.952 Å. Substrates having previously deposited films were repolished, to remove the film and to expose a fresh substrate surface, with either 0.05 μm alumina or colloidal silica suspensions. Both lead to epi-ready surfaces, but alumina-polished surfaces were slightly rougher than colloidal silica-polished ones (not shown), the latter of which had roughness values comparable to those of commercial surfaces.

A PLD chamber (Neocera) and KrF laser (λ = 248 nm) were used to deposit BSMO films. Substrates were cleaned in acetone and methanol for 10 min each and attached to the substrate heater using silver paint. Unless otherwise noted, deposition conditions were kept at 900 °C for the substrate temperature (*T*_s_), 2 mTorr for the total pressure (*P*_t_), 0.1% O_2_/99.9% N_2_ for the composition of the process gas (for a *P*_O_2__ = 2 × 10^–6^ Torr), 2 Hz for the laser frequency (*f*), and ≈1 J/cm^2^ for the laser energy density (*E*). These conditions are called the standard conditions. Parameters were changed individually from standard conditions to investigate the optimum deposition conditions. The 0.1% O_2_ gas mixture was used instead of pure O_2_ to control the oxygen partial pressure in the chamber without drastically changing plume dynamics (by fixing *P*_t_).

The deposition rate of BSMO on DSO (101)_o_ was determined by firing 1000 laser pulses and measuring the thickness of the film using X-ray reflectivity (XRR). The XRR pattern was fitted using the X’Pert Reflectivity software, and a rate of 0.012 nm/pulse was determined for standard conditions (see Supporting Information: S3 and Figure S1a). Deposited films ranged from approximately 11 to 66 nm in thickness according to the measured deposition rate. For the iPLD experiments, the number of pulses (rounded down to the nearest whole number) to deposit one layer of 3C BSMO was determined from the rPLD growth rate on DSO (101)_o_. 32 laser pulses (at 2 Hz over 16 s) were fired per cycle, followed by an annealing period of 1 min between each cycle. For some rPLD and iPLD films with roughnesses less than 4 nm, XRR was used to verify if the film thicknesses were as expected (see Supporting Information S3). All films were cooled at a rate of 10 °C/min under 200 Torr of the process gas, and they were not expected to be fully oxidized.

BSMO films were characterized by using XRD, atomic force microscopy (AFM), electron backscatter diffraction (EBSD), and high-angle annular dark field scanning transmission electron microscopy (HAADF-STEM). XRD scans were collected for the films on DSO (101)_o_ using a Panalytical X’Pert Pro MRD X-ray diffractometer, fitted with a polycapillary lens or high-resolution optics. AFM (NT-MDT Solver NEXT SPM) was used to image the surface morphology and measure the surface roughness of BSMO films deposited on DSO (101)_o_ single-crystal substrates.

EBSD patterns were captured in an FEI Quanta 200 scanning electron microscope using the TSL EBSD data collection software by EDAX. In brief, a high-energy electron beam (typically 10–20 keV) is accelerated toward a sample tilted at 70°. Elastically backscattered electrons (BSEs) that have undergone coherent Bragg scattering leave the crystalline sample and their diffraction pattern is recorded at the detector as an image.^42^ EBSD patterns contain the full 3D orientation of the local crystal relative to the experimental reference frame, in contrast to the typical pattern collected from XRD, which represents an average orientation over the entire sample in the direction of film growth (the orientation parallel to the substrate orientation). One disadvantage of EBSD for thin film applications is that the BSEs originate from 10 to 40 nm below the surface of the film, depending on the atomic number of the material and the accelerating voltage.^43^ This poses difficulties when the thickness is such that the substrate contributes to the pattern or if more than one phase or orientation exists within the probed volume of the film, as indexation of multiple phases is significantly more difficult and is not routinely possible now. EBSD characterization details, examples of EBSD patterns from epitaxial films, and descriptions of orientation relationships (ORs) are given in prior work.^40,41,44−48^

All EBSD patterns were indexed using a dictionary indexing (DI) method included in the open-source software package *EMsoft*. In DI, each pattern is compared to dictionaries of simulated patterns over all of orientation and expected phase space (e.g., 3C and 4H).^40,49,50^ The pattern with an orientation and phase that yields the highest dot product (similarity) is chosen as the answer for a given pattern. A longer description of DI and specifically of differentiating simulated EBSD patterns from 3C and 4H perovskite polymorphs are given by Zhou et al.^40^ and in Supporting Information S2.2. Automated DI is done for every scanned pattern, and maps of the scanned area showing phase and orientation are generated. EBSD orientation maps were constructed using the orientation imaging microscopy (OIM) analysis software by EDAX.

HAADF-STEM imaging was performed using a Thermo Fisher Themis 200 aberration-corrected scanning transmission electron microscope. The microscope was operated at 200 keV with a probe convergence semiangle of 17.9 mrad, and the inner detection angle of the HAADF detector was 69 mrad. Sequential image pairs were collected with orthogonal fast-scan directions, and a corrected image was formed from these using an open-source Python implementation of the nonlinear drift correction algorithm described by Ophus et al.^51^ Imaging was carried out for the 40 nm iPLD film on DSO (101)_o_. Prior to STEM sample preparation, the film was annealed in a box furnace under atmospheric pressure at 400 °C for 4 h to oxygenate the film. XRD and EBSD were carried out on the postannealed film and showed no change in the film peak position or phase. STEM samples were prepared via wedge polishing, followed by low-energy (0.5–2 keV) ion polishing in a Gatan precision ion polishing system (PIPS II).

## 3 Results

### 3.1 Regular PLD on Single-Crystal DyScO_3_

**Figure 1:**
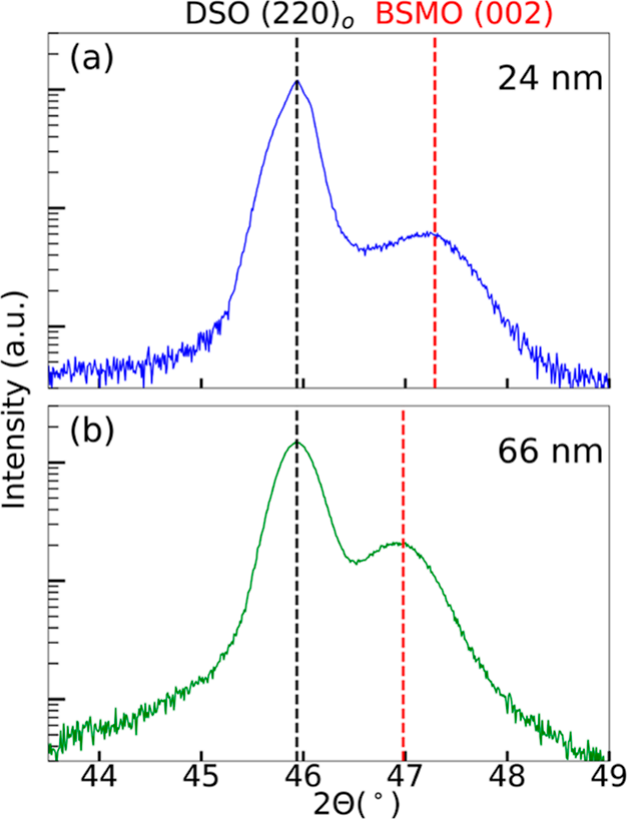
XRD patterns around the (202)_o_ reflection in DSO for (a) 24 and (b) 66 nm BSMO film deposited under standard conditions using rPLD. Vertical black and red dashed lines denote the location of the substrate and film peaks, respectively.

Approximately 24 and 66 nm BSMO films were grown on alumina-polished DSO (101)_o_ by using rPLD under standard conditions. The XRD patterns, registered around DSO (202)_o_, are shown in Figure 1a,b, respectively, for the 24 and 66 nm films. The peaks shown are the only out-of-plane peaks differentiable from the substrate peaks. The (202)_o_ substrate peaks are located at a 2θ position of 45.94° (*c*_pc_ = 2*d*_202_ = 3.949 Å), as expected. The film peaks are located at 47.29° (*c* = 3.842 Å) and 46.98° (*c* = 3.866 Å) for the 24 and 66 nm film, respectively. The unstrained, stoichiometric bulk 3C (002)_pc_ peak is projected to be at 46.75° (*c* = 3.883 Å), while the bulk 4H (023)_h_ peak is expected to be at 48.13°. Another film peak for the 4H (034)_h_, related to EBSD assignments,^40^ is also possible, and its relation to the (023)_h_ is discussed in Supporting Information S4. The experimental film peaks in Figure 1a,b are both indexed as the 3C phase, although it is difficult to establish whether or not the 4H phase is present in the film using out-of-plane XRD alone (see Supporting Information S4). The decrease in the 2θ peak position (increase in the lattice parameter) from Figure 1a,b indicates that the 66 nm film is more relaxed than the 24 nm film. On DSO, the 3C BSMO film is expected to be under in-plane tensile strain, so the out-of-plane lattice parameter is expected to be smaller (larger 2θ) for thinner, more strained films and larger (smaller 2θ) for thicker, more relaxed films.

**Figure 2:**
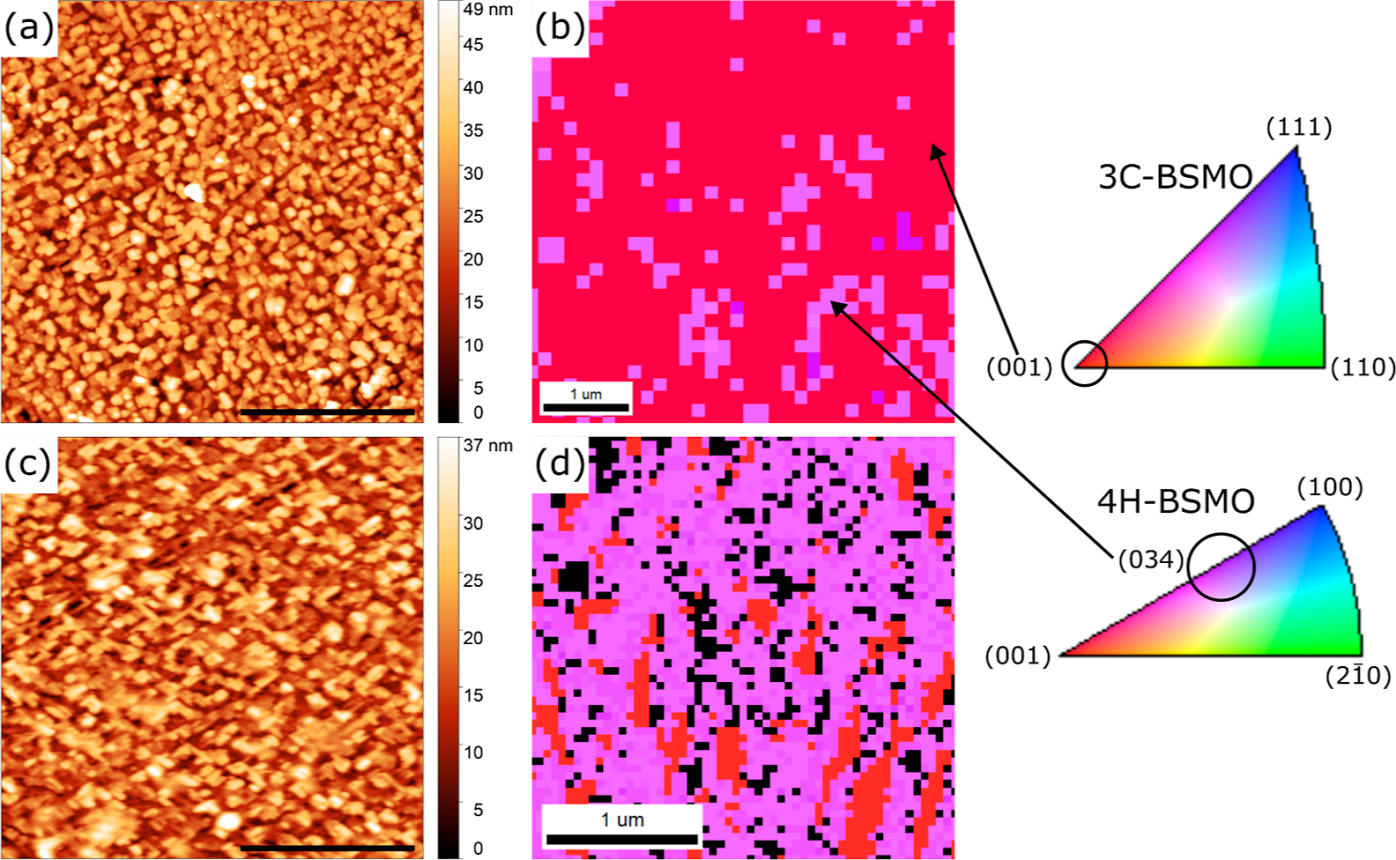
AFM images (a,c) and EBSD orientation maps (b,d) for the 24 (a,b) and 66 nm (c,d) BSMO films deposited under standard conditions using rPLD. Scale bars in the AFM images are 2 μm.

AFM images and EBSD orientation maps for the same 24 and 66 nm films are shown in Figure 2. The root-mean-square (rms) roughness for the AFM image from the 24 nm film in Figure 2a is 8.34 nm. The surface morphology comprises densely populated islands, some of which also coalesce, retaining a rough surface. This shows that the growth mode for BSMO under standard conditions is island growth. The average island height measured from two arbitrarily placed horizontal line scans (not shown) for the 24 nm film is 13.6 ± 6.93 nm, yielding a range of 27.8 to 85.5% of the nominal film thickness. EBSD orientation maps are shown in the right column of Figure 2 and color keys are displayed immediately to the right. Red areas were indexed as near (001)_c_ 3C regions and the purple areas were indexed as near (034)_h_ 4H regions, both with some variation due to orientation assignment that was less than 5°. The EBSD results in Figure 2b for the 24 nm film agree with the assignment of the XRD peaks to the 3C phase and with the absence of 4H XRD peaks owing to the (034)_h_ orientation of the 4H phase (see Supporting Information S4). The near-surface phase fractions, as indexed by DI, are 83.3% for 3C BSMO and 16.7% for 4H BSMO. Together, the XRD and EBSD results support the idea that 3C Ba_0.6_Sr_0.4_MnO_3_ has been stabilized using rPLD under standard conditions, though not as a pure phase.

The rms roughness for the 66 nm film, whose AFM image is given in Figure 2c, is 6.10 nm. The average island height is 8.30 nm ± 4.06 nm, measured from two arbitrarily placed horizontal lines (also not shown). The range of island heights is 6.42 to 18.7% of the nominal film thickness. These values are significantly less than that for the islands from the 24 nm film. Here, the decreased roughness compared to the 24 nm film may be a result of coalesced islands, a change in growth mode, or a change in phase formation. Regardless, the film still retains a relatively rough surface for the rPLD film, with a cross-hatched pattern suggestive of 4H formation in the near-surface regions, similar to what was seen for hexagonal BaRuO_3_ on cubic SrTiO_3_ (001)_c_.^52^ The EBSD orientation map for the 66 nm film is shown in Figure 2d. The phase fractions for the near-surface region of this film are 15.2% for 3C (red) and 69.7% (purple) for the 4H phase (with the balance of poorly indexed pixels shown in black). The increased fraction of 4H in the outer regions of this film is consistent with the observation of cross-hatch patterns from the AFM image in Figure 2c. In all experiments, this cross-hatch pattern correlated strongly with 4H formation, as determined by EBSD.

**Figure 3:**
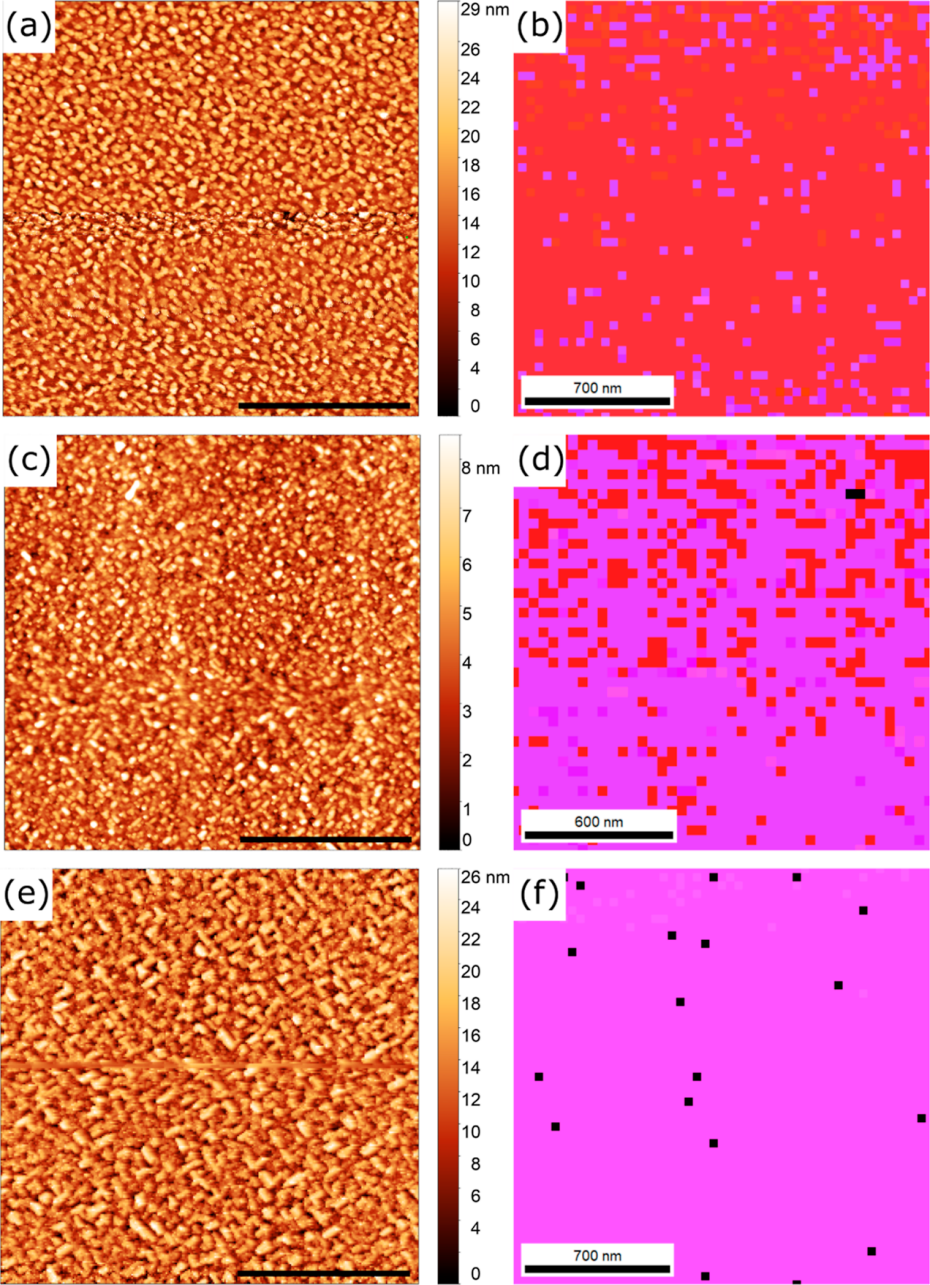
AFM images (a,c,e) and EBSD orientation maps (b,d,f) for 24 nm films with the following growth conditions: standard growth conditions (a,b), *f* = 20 Hz (c,d), and *E* = 2.2 J/cm^2^ (e,f). Scale bars in AFM images are 2 μm.

Positing that the rough island growth may correlate with a decreased nucleation probability of the metastable 3C phase, where rough surfaces present a wider range of nucleation/attachment sites, growth parameters were varied to produce flatter films. Figure 3 shows the AFM images and EBSD orientation maps of three 24 nm films deposited under different conditions. First, a 24 nm thick film was deposited under standard conditions on a colloidal silica-polished DSO (101)_o_ substrate. Compared to the 24 nm film in Figure 2a that was deposited on an alumina-polished substrate, the film surface shown in Figure 3a is smoother, with an rms roughness of 4.63 nm compared to 8.34 nm. Islands are generally smaller in-plane, and average heights are 6.72 ± 3.36 nm. No cross-hatch patterns are observed, whereas their formation can be seen in Figure 2a. Overall, the surface morphology of the colloidal silica-polished film is more homogeneous, indicating better control of the substrate surface quality than before. In the EBSD orientation map in Figure 3b, the 3C phase fraction is slightly higher than before in Figure 2b (90.3% 3C and 9.7% 4H compared to 83.3% 3C and 16.7% 4H). It should be noted that the scanned area and step size between the two orientation maps are quite different, but the results are still consistent with the difference in the surface morphology presented in the AFM image. The differences between these films are attributed to the preparation and roughness of the DSO substrate surface, which is improved by using colloidal silica suspensions as opposed to alumina suspensions. This observation is consistent with improved 3C nucleation on smoother surfaces, though island growth still predominated the AFM morphology. All substrates onward were therefore polished using colloidal silica.

Figure 3c,d is from a 24 nm film deposited under nearly standard conditions with the exception that the laser frequency (*f*) was increased 10-fold, from 2 to 20 Hz. Such an increase in *f* is expected to decrease roughness owing to kinetic effects, and this was found to be true. The rms roughness in Figure 3c is decreased to 1.30 nm compared to 4.63 nm in Figure 3a. The increased *f* consequently decreases (increases) the fraction of 3C (4H) in the EBSD characterization of the film to 21.6% (78.1%), as shown in Figure 3c. It should be noted that there was significant charging on the surface of this film, resulting in a vertical drift during the EBSD scan. Therefore, the vertical scale is smaller than the horizontal one. That the morphology of the film in Figure 3c is largely island-like and that the film is mostly 4H indicates that the film is transitioning from 3C to 4H. Overall, the increased kinetics owing to increased *f* not only decreased roughness but also decreased the nucleation advantage of the metastable phase.

Following this, a film was deposited under nearly standard conditions, but the laser energy density (*E*) was increased from 1 to 2.2 J/cm^2^, which increases the instantaneous arrival rate per pulse (the growth rate was not determined). AFM and EBSD characterizations of this film are shown in Figure 3e,f. The rms roughness is approximately the same as for the film grown under standard conditions (4.28 nm vs 4.63 nm), but the morphology is entirely cross-hatched patterns, making the comparison somewhat indirect. In agreement with the cross-hatch morphology, the entire film is indexed as 4H in the EBSD analysis.

The results described with respect to Figure 3 indicate that the control over the crystallization (nucleation or attachment) stage has important consequences for the outcome of the phase competition between the individual epitaxial variants of the two polymorphs. Decreased static roughness (via substrate preparation) increases selection of the metastable phase, while decreased kinetic roughness (via increased *f*) does not overall. Increased supersaturation (via increased *f* and *E*), which increases rates and probabilities for both competing nucleation possibilities, increases the fraction of stable 4H in the film.

### 3.2 Interval PLD on Single-Crystal DyScO_3_

**Figure 4:**
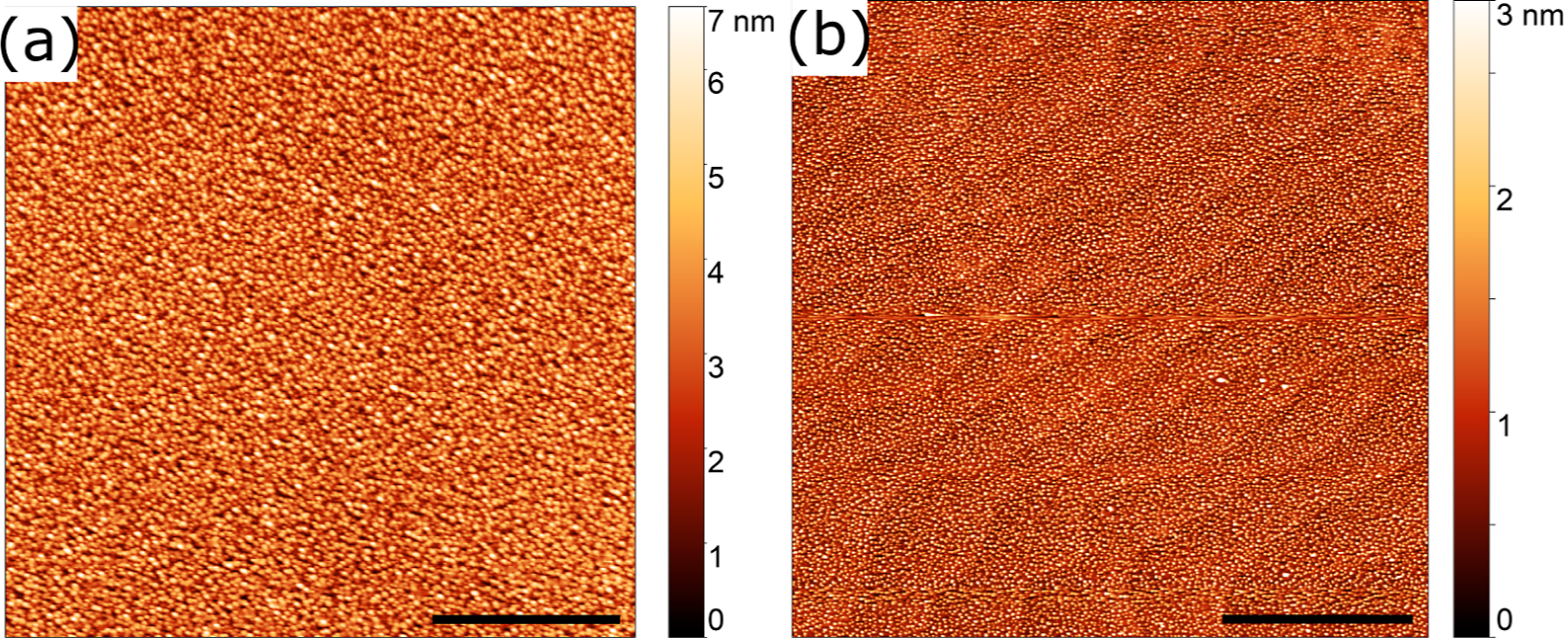
AFM images comparing (a) 11 nm rPLD film vs (b) 15 nm iPLD film. Scale bars are 0.2 μm.

As an alternate approach to attain flat films and influence nucleation, films targeted to be approximately 12 nm thick (approximately 31 3C BSMO unit cells, with some error due to rounding the number of pulses per unit cell) were deposited under standard conditions using rPLD and iPLD (as described in the Experimental Section). A side-by-side comparison of the surface morphology between the rPLD and iPLD films is shown in Figure 4a,b, respectively. The rPLD film has the typical dense island surface morphology, while the iPLD film maintains the step and terrace structure from the DSO substrate. This difference in the surface morphology indicates that the LBL growth can be obtained with iPLD, with small islands on the terraces indicating terrace nucleation and coalescence as the LBL growth mode. The rms roughness values for the rPLD and iPLD films were 1.07 and 0.550 nm, respectively. Due to the low roughnesses, film thickness was determined using XRR (see Supporting Information S3 for fitted simulations). The rPLD and iPLD films were 11 (Figure S1b) and 15 nm (Figure S1c) thick, respectively. The difference in the measured thickness may arise from the different roughnesses: the coherent XRR signal is likely from the uniform dense layer below the rough surface (which contributes to noise) in the rPLD film, which then underestimates the total deposit thickness, while that in the smoother iPLD film is closer to the total thickness. There may also be some variability in deposition from growth to growth that accounts for some of the difference but not likely the overall ≈30%. It should be noted that the computation of 32 pulses per monolayer came from rPLD films, which led to deposition of more than one monolayer per iPLD cycle (by approximately 25%). Overall, though, interrupting PLD growth did afford time for the deposit (islands) to relax such that they coalesced and recovered much of the initial morphology of the surface, as described in prior iPLD reports.^33,37,53,54^

**Figure 5:**
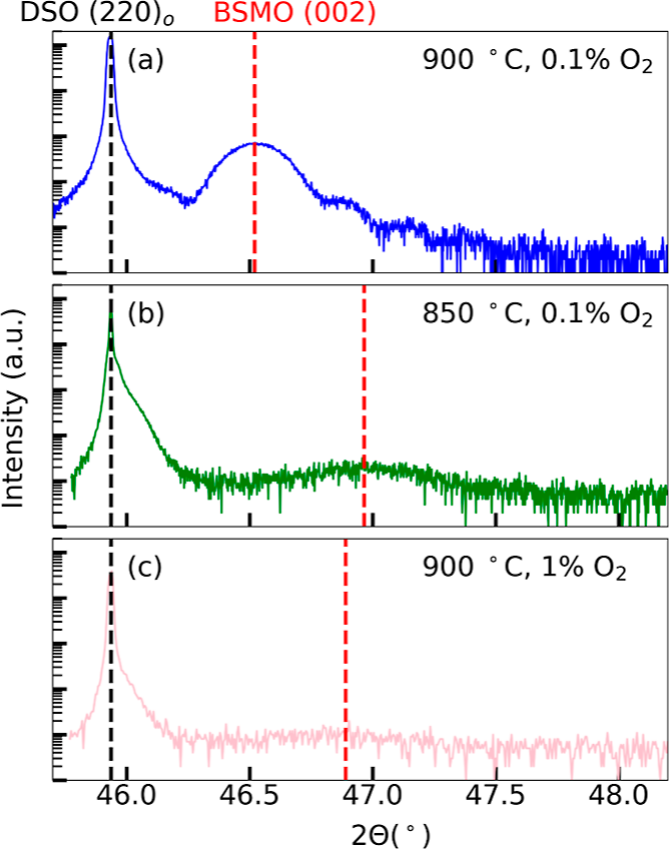
XRD (high-resolution optics) patterns around the (202)_o_ reflection in DSO of 40 nm iPLD films at different conditions: (a) standard conditions, (b) *T*_s_ = 850 °C, and (c) *P*_t_ = 2 mTorr 1% O_2_ (*P*_O_2__ = 2 × 10^–5^ Torr). The red (black) vertical lines in these images are at the same angular location for the films (substrates) as those in Figure S5a–c.

Three films, each approximately 40 nm thick, were deposited by iPLD using different deposition conditions to explore the thermodynamic effects on phase formation. XRD scans from each, registered using high-resolution optics to resolve the film from the substrate more readily, are shown in Figure 5. The pattern from the 40 nm film deposited under standard conditions is shown in Figure 5a, and it corresponds to the 3C structure (no evidence of 4H was found in the pattern). The presence of Laue oscillations from the film indicates that the 3C film is coherent and relatively flat, even at this thickness, and has good crystallinity.^55^ XRR analysis estimates this film to be around 41 nm (see Figure S1d), again suggesting that the number of pulses used in the iPLD cycle overshot the deposition of precisely one monolayer (in this case by few percent). The 2θ position of the film peak is located at 46.52°, which corresponds to the largest out-of-plane lattice parameter of all of the films in this work, at *c* = 3.902 Å. This suggests that, under standard conditions and using iPLD, the film in Figure 5a has a higher concentration of oxygen vacancies (i.e., a higher concentration of Mn^3+^ ions) than that expected from a stoichiometric film whose lattice parameter would be *c* = 3.883 Å from the Vegard’s law approximation.^8^

Films were also deposited in nearly standard conditions with either the substrate temperature being lowered from 900 to 850 °C (see Figure 5b) or the process gas being changed from 0.1 to 1% O_2_ (i.e., in a higher oxygen partial pressure, see Figure 5c). Both of these conditions tend to decrease the stability of the 3C phase. It is immediately noticeable that the intensities of the film peaks are much lower and broader than that of Figure 5a. Langenberg et al.^14^ attributed such a decrease in X-ray intensity to the increased formation of the 4H phase in BSMO (*x* ≤ 0.5) films at lower temperatures and higher oxygen partial pressures, or poor crystal quality. High-resolution optics in thin film XRD are more sensitive to the crystal quality in the film than polycapillary lens optics; therefore, films with low crystal quality tend to have low-intensity peaks. A weak shoulder to the high-angle side of the DSO substrate peak (see Figure 5b,c) is sometimes present in repolished DSO substrates with no films. The origin of these shoulders could be from residual subsurface damage induced by the repolishing method. XRD patterns from the same three films but using the polycapillary lens optics are given in Supporting Information S5, Figure S3, in which the peaks for the latter two films are more visible than in Figure 5b,c. The film peak in Figure 5b is located at 46.96° (*c* = 3.868 Å). Although the film peak is barely visible in Figure 5c using the high-resolution optics, the 2θ position is located at 46.89° (*c* = 3.873 Å) according to the XRD pattern using the polycapillary lens optics (see Figure S3e). However, there is some error associated with the film peak position, as measured from the polycapillary lens optics compared to the high-resolution optics due to beam divergence and higher background noise.

**Figure 6:**
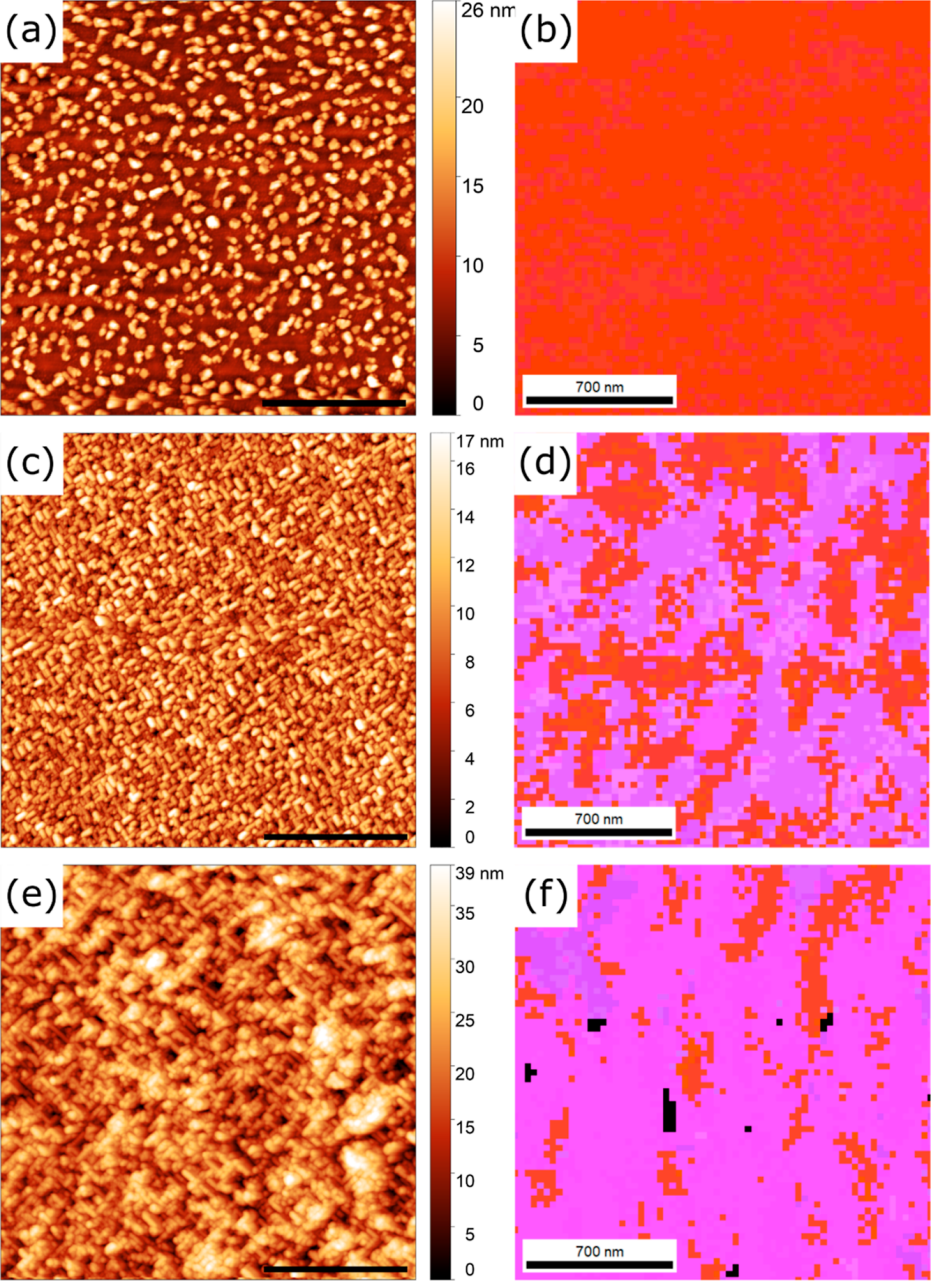
AFM images (a,c,e) and EBSD orientation maps (b,d,f) of 40 nm iPLD films deposited under different conditions: (a,b) standard conditions; (c,d) *T*_s_ = 850 °C; (e,f) *P*_t_ = 2 mTorr 1% O_2_ (*P*_O_2__ = 2 × 10^–5^ Torr). AFM scale bars are 2 μm.

The AFM images and EBSD orientation maps for these same three iPLD films are shown in Figure 6. For the film deposited under standard conditions, whose AFM image is given in Figure 6a, the surface has sparse islands that are separated by smoother film regions, although no surface steps are evident. This growth is consistent with that described for the film shown in Figure 4b, and with the overshoot of the thickness described previously. It is likely that the terrace-nucleation and coalescence LBL growth mode leads to coarsening of islands over time. The rms roughness measured by AFM is 3.79 nm, which is already smoother than that of the 24 nm rPLD film in Figure 3a. The 41 nm thickness measured from XRR (see Figure S1d) likely comes from the smooth underlayer and not from the surface islands that produce the 3.79 nm rms roughness, which is consistent with the thickness value being closer to that of the rPLD-calibrated target thickness. In Figure 6b, the entire film was indexed as 3C BSMO (001)_c_, consistent with the XRD results from Figure 5a and the AFM surface morphology. In-plane orientation maps are shown in Figure S6, and orientations are near (100) and (010), indicating that epitaxial growth exists in all three directions. The epitaxy can be described as [110]_pc_(001)_pc_ ∥ , for the pseudocubic (pc) BSMO film and orthorhombic (o) DSO substrate. Additionally, a kernel average misorientation (KAM) map was constructed from the EBSD data for this film and is shown in Figure S6. The low average misorientation (0.85°) further supports that epitaxial growth was achieved for the 3C film. Between the XRD and EBSD analyses, this iPLD film can be considered single-phase 3C at a thickness nearly double the rPLD film with the highest phase fraction in EBSD.

For the iPLD film deposited at 850 °C, whose AFM image is shown in Figure 6c, the whole surface consists of the 4H cross-hatch pattern, but the rms roughness is only 2.96 nm. The smoother surface may be related to the decreased thermal energy available for adatom diffusion/cluster rearrangement. In the orientation map shown in Figure 6d, the fraction of 3C drops to 40.6%, while the fraction of 4H is 59.4%. The cross-hatch pattern from the AFM image agrees with the EBSD analysis for these films (Figure 6d), keeping in mind that the EBSD signal comes from tens of nanometers, so regions deeper than those contributing to AFM surface morphology may index as 3C.

Figure 6e is the AFM image of the iPLD film deposited by using 1% O_2_. The cross-hatch pattern from the epitaxial 4H polymorph is visible and the rms roughness is 6.50 nm, which is comparable to the 66 nm film shown in Figure 2d. The increase in surface roughness in the film deposited at higher oxygen pressures is consistent with a destabilization of the 3C phase at higher pressures and an overall rougher growth for the 4H polytype. In the EBSD orientation map in Figure 6f, the fraction of the film that is indexed as 3C (4H) in EBSD greatly decreases (increases) to 14.5% (84.8%). This low volume fraction for 3C is consistent with the low-intensity film peak from its XRD pattern in Figure 5c. Whether changes to the iPLD cycle could increase the 3C stability was not explored. Overall, these observations from the three iPLD films illustrate that a combination of thermodynamic (standard growth conditions) and kinetic (iPLD) factors controls the persistent nucleation of the metastable 3C phase at the growth surface throughout deposition. Surface morphologies and orientation maps are consistent with their XRD patterns in Figure 5a–c and support the idea that standard conditions are optimal for the stabilization of 3C BSMO using iPLD.

**Figure 7:**
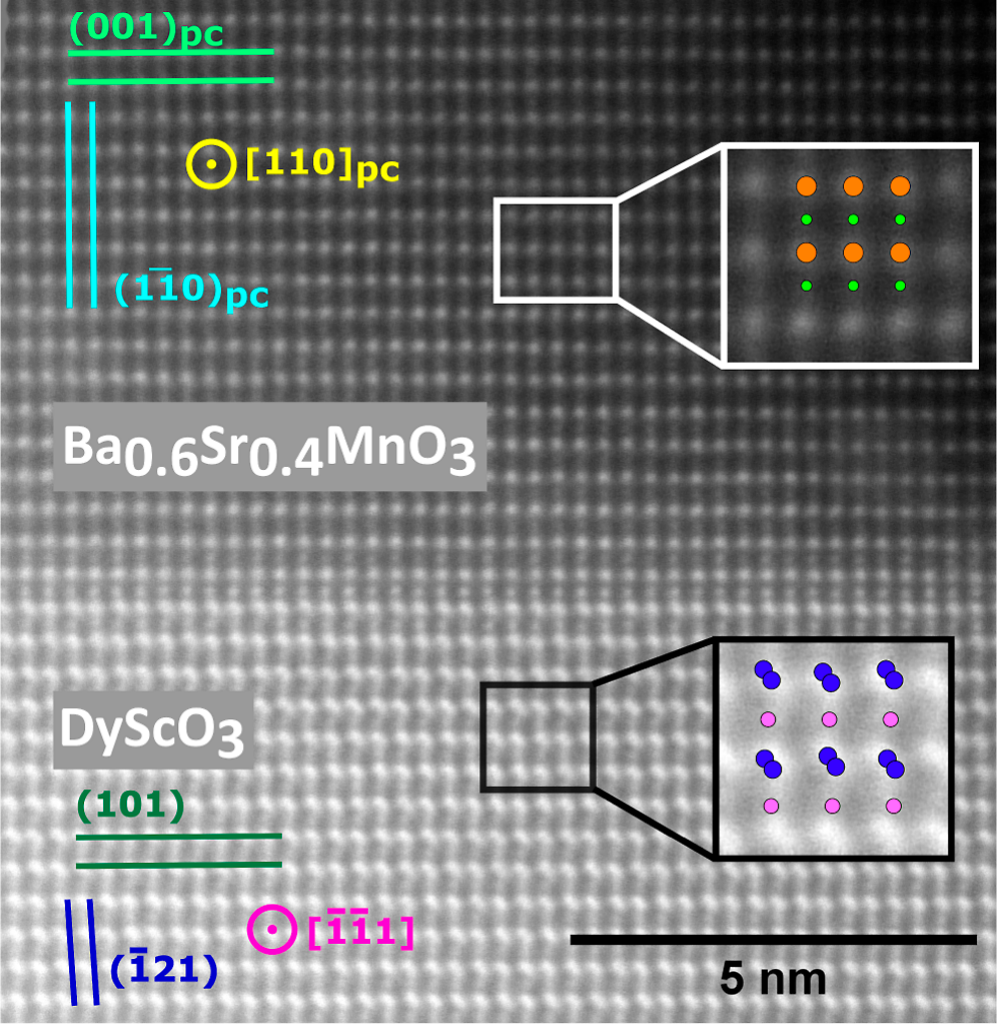
High-magnification HAADF-STEM image of a [110]_pc_ cross-section of the 40 nm iPLD film deposited under the standard conditions. Insets show the atomic alignment with the theoretical 3C BSMO and DSO crystal structures. Orange, green, blue, and purple circles are Ba/Sr, Mn, Dy, and Sc atoms. Planar alignments and viewing directions are notated in the figure on the left side.

**Figure 8:**
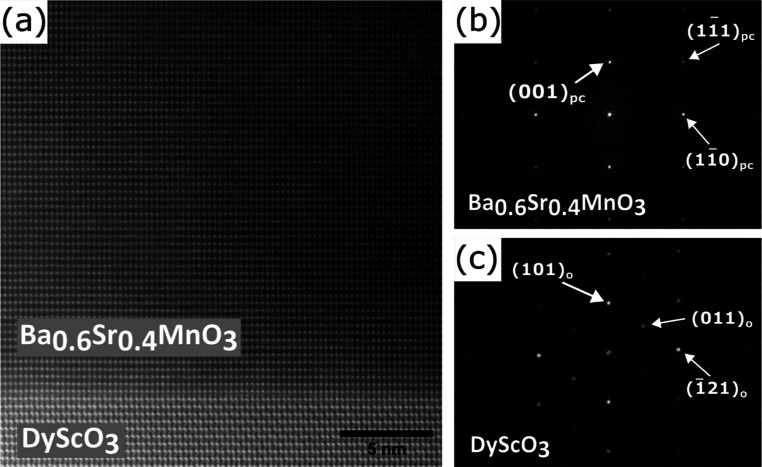
(a) Low-magnification HAADF-STEM image of a [110]_pc_ cross-section of the 40 nm iPLD film deposited under standard conditions. (b,c) FFTs of regions originating from the film and substrate, respectively. The kinematically forbidden spots at (11̅0)_o_ and (1̅10)_o_ appearing in (c) are visible due to double diffraction effects.

HAADF-STEM was used to examine the real-space atomic structure of the single-phase, ≈40 nm thick films of the highly metastable 3C Ba_0.6_Sr_0.4_MnO_3_ fabricated using iPLD in standard growth conditions. HAADF-STEM images were collected for a [110]_pc_ cross-section of the optimized iPLD film and are shown in Figures 7 and 8. Figure 7 highlights the interface between the DSO (101)_o_ substrate and the epitaxial (001)_pc_ BSMO film. The good coherent alignment between the substrate and film lattice planes along the growth direction (the vertical direction in the image) is typical of films with high crystalline quality. The insets from the film and substrate show the atomic alignment between the image and ideal crystal structures (modeled by CrystalMaker).^56^ We find that [110]_pc_ ∥ []_o_ (the imaged zone axes) and (001)_pc_ ∥ (101)_o_ parallel to the interface, as indicated in the figure.

A lower magnification image from a different area of the iPLD film is shown in Figure 8a, showing a much larger region of the film. Even over this larger region, the film is defect (dislocation) free. Based on the lattice mismatch with the substrate (1.80%) and high Ba content, the BSMO film should relax above 10 nm,^14^ though this image contains 20 nm of the film thickness of the 41 nm thick film. The out-of-plane lattice parameter was directly measured as 3.926 Å, which is within the error (0.615%) of the average lattice parameter measured from XRD. The in-plane parameter was 2.785 Å, which is 1.50% larger than the predicted *d*-spacing for (110)_c_ and indicates that the film is under tensile strain in the plane.

The FFTs of large regions of the film and substrate are shown in Figure 8b and c, respectively. These patterns are consistent with 3C BSMO growing on o-perovskite DSO. No sign of the 4H phase was found anywhere in the film region using the FFT. The only defects found elsewhere in the film were a grain boundary and twin boundaries (see Supporting Information S6 and Figure S4(a,b)) oriented similarly to the Ba_0.4_Sr_0.6_MnO_3_ twin boundaries found by Langenberg et al.^14^ However, when carrying out the FFT of regions within a domain, we found no evidence of any secondary phase (see Figure S4(c)). These TEM results support the conclusions made from XRD and EBSD data: iPLD Ba_0.6_Sr_0.4_MnO_3_ films grown under standard conditions are single phases with high crystalline quality with thicknesses of more than 40 nm.

## 4 Discussion

The results presented herein show that the epitaxial phase boundary of the metastable 3C polytype in Ba_*x*_Sr_1–*x*_MnO_3_ was pushed to a value of (at least) *x* = 0.6 on DSO (101)_o_. This was achieved at a relatively high temperature (900 °C) and in a controlled total (*P*_t_ = 2 × 10^–3^ Torr) and effective oxygen partial pressure (*P*_O_2__ = 2 × 10^–6^ Torr) environment using rPLD. Furthermore, the results indicate that the persistent nucleation of the metastable 3C polytype during continued deposition limits the thickness to which one can fabricate a phase-pure film with rPLD. Films deposited with iPLD in otherwise identical process conditions to rPLD were single-phase 3C films up to at least 40 nm, indicating that no obvious thermodynamic critical thickness limits phase stability, persistent nucleation of such highly metastable films is possible, and compositions of BSMO with *x* > 0.6 are likely attainable. Here, we discuss these observations further.

### 4.1 Thermodynamic Thickness Limits to Metastable 3C BSMO

In general, epitaxial stabilization of a bulk metastable phase occurs because there is a change in thermodynamic stability during nucleation, primarily arising from the low-energy (high-energy) interface between the metastable (stable) phase and a substrate or pre-existing deposit.^20−26^ In addition to the area-specific interface energy, there is the thickness-dependent volumetric (bulk and epitaxial strain) energy. Xu et al.^24^ and Zhou et al.^26^ have shown through a first-principle investigation that, respectively, for TiO_2_ and (Ba/Sr)MnO_3_ polymorphs, epitaxial stability on differently oriented (Ba/Sr)TiO_3_ substrates can accurately reproduce experimental trends by including an interface energy term along with the bulk formation and strain energies. The stability of the metastable phase is therefore thickness dependent, and there is a crossover point at which the bulk stable phase becomes the lower energy phase for the entire film. It was computed that for under 2 stoichiometric layers of BaMnO_3_, 3C could be epitaxially more stable than 4H.^26^ It is important to emphasize that this energetic preference refers to the preference for the entire pre-existing film to transform to the more stable phase; it does not refer to the instantaneous crystallization of new adatoms during continued growth. It can be readily shown that this back-conversion is a low-probability event^22^ and there is no evidence in the literature that this crossover leads to the phase transformation it predicts in systems that require solid-phase nucleation and growth. The existence of mixed-phase regions whose relative phase fractions change with growth is not consistent with a solid-phase transformation governed by the thickness-dependent preferences of the previously crystallized film.

Using similar thermodynamic arguments, the epitaxial strain of a coherent metastable (or stable) film is expected to relax via dislocations at some thickness.^57^ After strain relaxation, the metastable phase nuclei become slightly more preferred as the strain energy penalty drops to zero. On the other hand, there is very little change in the strain energy penalty (assuming it is near zero initially) with respect to the relaxed stable phase due to its incoherent interface.^26^ This is tantamount to assert that the ideal substrate for epitaxial stabilization is the target relaxed metastable material itself. Thus, thickness-dependent strain relaxation by itself cannot account for the observation that 4H films in rPLD occur at relatively low thickness values. This is supported by the observation that regions of the 3C phase persist to the near-surface in the 66 nm film deposited by rPLD, and those regions of the film have relaxed.

To grow thick films of a metastable material, a persistent nucleation preference is required throughout the growth of the film. In the ideal epitaxial growth experiment, for systems without solid-phase nucleation back-transformation pathways, every controlled nucleation event would be identical and reproduce the preference for the metastable phase,^20−26^ allowing for films to be grown to arbitrary thicknesses. However, unless the energetic preference is absolute (that nucleation of the competing phase is not possible), there is some finite probability that the stable phase will nucleate. Thus, nucleation should occur in a stochastic fashion such that, after some number of nucleation events, or the thickness of the film, stable phase nucleation should occur.^58^ This particular thermodynamic limit is affected by the effective supersaturation, which is related to the number of atoms crystallizing (related to *f* and *E*), temperature, and oxygen pressure (which affect bulk stability).^22,58−60^ Once a region of the more stable phase nucleates, e.g., 4H in BSMO, the probability of its nucleation in that region immediately becomes preferred (possibly absolute), due to both its inherent bulk stability and its local epitaxial stability upon the preexisting stable phase deposit.^20−26,58^ From that thickness onward, the local probabilities of nucleation vary with respect to location depending on the structure of the pre-existing film in the local area,^58^ and ultimately the film will convert completely to the stable (4H) phase with increased thickness, with some thickness range of mixed phase. That 40 nm films can be fabricated using iPLD indicates that any single-phase thickness value in rPLD is likely related to a thickness-dependent change in local nucleation probabilities, in a manner that decreases the preference for the epitaxially stabilized metastable phase.^58−61^

### 4.2 Roughness Effects on Nucleation of 3C BSMO

The main difference between rPLD and iPLD films deposited under standard conditions is simply the annealing period after the deposition of approximately one monolayer. Here, the annealing period is 3.75 times longer (1 min) than the deposition period (16 s). During the annealing period, various relaxation processes can occur. The most prominent outcome of these relaxations is the improved flatness of the films, as reported elsewhere for iPLD.^33,37,53,54^ In rPLD, the morphology of the film indicates a dense island growth mode and their coalescence, with the overall roughness increasing with thickness. In iPLD, the relaxation time allows for the dense islands to flatten and coalesce, decreasing the relative roughness and even leading to LBL growth. Because the deposition was not exactly a monolayer, there were some residual islands on the terraces that appeared to coarsen over time (with thickness). Importantly, these relaxations also improved the outcome of nucleation to be the 3C metastable phase.

Several thermodynamic possibilities that may destabilize 3C nucleation can be related to the roughness. As described above, the metastable 3C nuclei are more stable at lower thicknesses and 4H at higher thicknesses,^26^ but the crossover thickness value is not known exactly. If this value is close to the island height in rPLD films and the islands can reconfigure between the two phases (which a dense coherent layer cannot do), then higher islands favor the stable 4H phase. iPLD allows for the islands to flatten and remain below such a critical value (if important) during each layer deposition. As the growth surface becomes flatter and more homogeneous, the probability that the more stable phase nucleates at heterogeneous islands diminishes.

Additionally, local misorientations (or other extended defects) can form as rough islands coalesce. We show in Supporting Information S8 (Figure S7) the difference in local misorientation between an rPLD and an iPLD film of BSMO (for *x* = 0.5) deposited under standard conditions. While both films were indexed as 3C, the rPLD film shows a higher degree of misorientation throughout the surface of the film compared to the iPLD film, where there is virtually no misorientation except for near islands (and possibly a twin boundary). Such extended defects may provide sites for stable phase nucleation^59−61^ and likely accumulate throughout rPLD growth. iPLD allows time for local misorientations (or other extended defects) to recover within each thin monolayer, avoiding their accumulation that occurs in rPLD films, and this recovery would continually reset nucleation probabilities in favor of persistent 3C crystallization.

Another possibility related to roughness affects kinetics. Rough surfaces tend to have lower surface diffusivities of adatoms.^62^ A lower diffusivity prevents adatoms from accessing the global minimum in energy and assists in the formation of higher energy events that may not otherwise form or persist if adatoms were to access lower energy sites. This reasoning could explain the increased presence of the 4H phase with thickness in the rPLD films (Figures 1 and 2). However, from the depositions in which process parameters were varied to achieve kinetically flat films (Figure 3), 4H was favored, even when flatter films were achieved. This observation supports the notion that the 4H phase has a kinetic preference in formation. From this perspective, flatter films in iPLD improve surface diffusion and disfavor kinetic trapping^63^ of 4H material. Other kinetic effects are described next.

### 4.3 Structural Relaxations in iPLD and Stabilization of 3C BSMO

Higher temperatures and lower oxygen pressures favor the 3C phase over the 4H phase, largely because they increase the oxygen vacancy concentration.^12,29,30,64−66^ Compositions with lower Ba contents also favor the 3C phase over the 4H phase, stabilizing corner-sharing Mn–O octahedra over face-sharing ones.^67,68^ When adatoms (or clusters or nuclei) achieve equilibrium, these structural preferences are controlled by the process variables *T* and *P*_O_2__ and the composition of the target. However, the instantaneous values can be considerably influenced by plasma conditions and kinetics. Before arriving at equilibrium, the adatoms and their clusters may have local values distinct from their equilibrium ones;^63^ if such clusters are not able to relax, they can be retained in the film and influence the outcome of nucleation.^63^ In iPLD, time is allowed for the outermost monolayer to rearrange sufficiently to impact roughness, which also indicates that structural relaxations may occur.

SrMnO_3_ films have been known to lose significant oxygen content postdeposition, even after annealing in high oxygen partial pressures,^28,69,70^ indicating that excess oxygen can be incorporated during rPLD, adding a destabilizing effect on the 3C phase. Excess oxygen can come from the target, being incorporated into the plasma and condensing material. If excess oxygen is lost during the relaxation time in iPLD, then regions locally favoring 4H are lost. The out-of-plane lattice parameter of the single-phase 40 nm film is the largest of all 3C films and suggests that it has the largest oxygen vacancy concentration (not measured). However, due to varying oxygen content with time, techniques such as X-ray photoelectron spectroscopy were not performed but may be of interest in future studies. The film is also largely unrelaxed at a relatively large thickness (see Figure 8), but differentiating whether the oxygen content is linked to deposition rates or lack of strain relaxation was not done. Also, the effects of varying temperature and pressure during iPLD lead to observations that are consistent with bulk phases and rPLD: lower temperatures and higher oxygen pressures favored the 4H phase, as shown in Figure 6b,c, indicating that the process variables do play an important role even in iPLD.

Because the 3C and 4H phases are in close competition, we expect clusters to naturally and dynamically access both face-sharing and some corner-sharing arrangements of the Mn–O octahedra. Based on the outcome of rPLD experiments in which kinetic effects were modified, increasing *f* or increasing *E*, the amount of 4H increased with decreased time between pulses or with more material arriving on each pulse, indicating that the formation of face-sharing (4H) clusters or nuclei is not kinetically hindered compared to corner-sharing ones, and they may even be favored over direct formation of all corner-sharing ones. In Ostwald’s step rule, the first phase that nucleates is not necessarily the most stable phase, but often a metastable phase with a lower activation barrier.^63,71^ If some face-sharing regions, which are metastable in the epitaxial condition presented here, get captured kinetically, then the structure will be impacted: infrequent events will lead to stacking faults (which are present in the iPLD film, see Supporting Information S6), while frequent events will lead to 4H phase stability (50% face sharing and 50% corner sharing) via the Ostwald step rule mechanism. Similar observations could be made if the local Ba content varies on arrival, as this would impact local phase stability. All of these kinetic issues, oxygen fluctuations, natural dynamic access of multiple low-energy states, or a low-energy barrier to 4H formation, can relax during the interruption in iPLD and assist in arriving at the lowest energy structure, which is 3C formation by epitaxial stabilization.

## 5 Conclusions

Ba_0.6_Sr_0.4_MnO_3_ films were epitaxially stabilized on DyScO_3_ (101)_o_ substrates, but the successful growth of a single-phase film to reasonable thickness values requires a careful balance of thermodynamics and kinetics, which are controlled through deposition parameters. In rPLD films, there is a thickness dependence on the fraction of the film that is 3C, which decreases progressively with thickness. Furthermore, low oxygen pressures and high substrate temperatures are expected to favor 3C, but films are rough and difficult to characterize. Increasing the laser frequency and energy density results in relatively smoother films, but the fraction of the 4H phase increases with these parameters, indicating that it is the kinetically preferred phase in nucleation. iPLD offers an avenue around the kinetic issues faced in rPLD, and single-phase 3C BSMO films were prepared up to 40 nm in thickness. By interrupting the otherwise identical rPLD after approximately each unit-cell monolayer, which provides the monolayer time to rearrange itself toward its lowest energy structure, both flatness and 3C phase fraction are significantly improved. These results suggest that a higher Ba-content BSMO film is possible, as growth issues associated with changing temperature and oxygen partial pressures can be overcome using iPLD. It is expected that the persistent nucleation of epitaxially stabilized films in iPLD may also be important for other material systems where there are difficulties in growing thick layers of single-phase films.

## Supporting Information Available

BSMO structural preferences, expanded details of experimental methods, including target preparation and dictionary indexing, XRR data, epitaxial 4H XRD challenges, XRD patterns using lens optics, HAADF-STEM evidence of defects in the iPLD film, maps of local epitaxy in three orthogonal directions, and local misorientation from two growth approaches (PDF)
