# Supplementary material for: Epitaxial Stabilization and Persistent Nucleation of the 3C Polymorph of Ba0.6Sr0.4MnO3
Source: ACS Appl Mater Interfaces. 2024 Jan 17;16(4):4873–85. doi: 10.1021/acsami.3c11934 (PMC10835664; doi:10.1021/acsami.3c11934)
Supplement: Supplementary file 1 — am3c11934_si_001.pdf [file am3c11934_si_001.pdf]

Supporting Information: Epitaxial Stabilization and Persistent Nucleation of the $3C$ Polymorph of $Ba_{0.6}Sr_{0.4}MnO_3$

Catherine Zhou, Charles Evans, Elizabeth C. Dickey, Gregory S. Rohrer, Paul A. Salvador*

Department of Materials Science and Engineering, Carnegie Mellon University, 5000 Forbes Ave., Pittsburgh, Pennsylvania 15213, USA.
*Corresponding author: *paulsalvador@cmu.edu*

December 17, 2023

Abstract

This document provides Supplementary Information to the main text. § S1 discusses the structural preferences of BSMO materials. § S2 gives expanded details of some experimental details, including target preparation (§ S2.1) and dictionary indexing (§ S2.2). § S3 provides information on how film thicknesses and growth rates were measured using x-ray reflectivity (XRR). § S4 discusses the possible XRD diffraction peaks from a film with $4H$ and why it is so difficult to observe them experimentally. § S5 provides x-ray diffraction patterns of the rPLD and iPLD films using x-ray lens optics that have lower resolution but higher intensity than the optics used to generate patterns shown in the main text. § S6 presents HAADF-STEM evidence of defects in the iPLD deposited coherent, single phase, 40 nm thick film of $Ba_{0.6}Sr_{0.4}MnO_3$ on orthorhombic (o) $DyScO_3$ $(101)_o$. § S7 gives the inverse pole figure maps along three orthogonal $\{100\}_{3C}$ directions, illustrating local epitaxy across the entire film. Finally, § S8 describes local misorientation analysis from $Ba_{0.5}Sr_{0.5}MnO_3$ films, note the composition change, deposited on $DyScO_3$ (DSO)$(101)_o$ substrates using iPLD and rPLD, which is consistent with increased internal relaxation during iPLD growth.

S1 Structural Preferences

The endpoints of the BSMO system, $SrMnO_3$ and $BaMnO_3$, are well studied with respect to their crystal structures. Because of the large alkaline earth cation size with respect to Mn^{4+}, the hexagonal phases are more energetically preferred over the cubic phase.[1] The ground state structure is often predicted using the Goldschmidt tolerance factor (t), which describes the relative bond lengths between A-O and B-O bonds, where A is the alkaline earth ion and B is the Mn ion.[2] $SrMnO_3$ and $BaMnO_3$ have t-values of 1.033 and 1.097, respectively, which makes their predicted ground state structure a hexagonal one because $t > 1$.[1,3] The ground state structure for $SrMnO_3$ is described as a four-layered hexagonal ($4H$) structure with alternating corner-sharing and face-sharing MnO_6 octahedra. At elevated temperatures (above 1400 °C), $SrMnO_3$ can transform into another polytype, the three-layered cubic structure ($3C$) with fully corner-sharing octahedra. It should be noted that the $3C$ notation only refers to the stacking type, as structures can be non-cubic (e.g. tetragonal or orthorhombic) with $3C$ stacking of the nearly close-packed (eutactic) planes. The ground state structure of $BaMnO_3$ is the two-layered hexagonal ($2H$) structure with completely face-sharing octahedra.[4] The high temperature structure is the $4H$ one described for $SrMnO_3$, for which the phase transition begins above 1550 °C. For these alkaline earth manganite systems, the ratio between Mn^{3+} and Mn^{4+} ions is related to the oxygen vacancy concentration, which increases with temperature. When the Mn^{3+}/Mn^{4+} ratio increases due to higher oxygen vacancy concentration, so does the average Mn-O bond length, bringing the tolerance factor closer to 1. However, Mn-O bond lengths vary less significantly with temperature than the A-O (A = Sr, Ba) bond lengths vary, and these differing thermal expansion of bond lengths complicate the prediction of tolerance factor without experimental studies.[5] Additionally, oxygen partial pressures also affect the temperature at which oxygen vacancies form, and subsequently the phase transition temperature.[6] Such dependence of structure on

temperature, oxygen partial pressure, and bond lengths are difficult to capture using density functional theory (DFT), and therefore structural predictions can benefit from experimental parameterization of the tolerance factor for similar materials.[5]

S2 Expanded Details of Experimental Methods

S2.1 Target Fabrication

Stoichiometric amounts of $BaCO_3$, $SrCO_3$, and MnO_2 were mixed in ethanol, ball milled wet overnight, and subsequently pressed uniaxially in a stainless-steel die (24 mm diameter) at pressures >10,000 psi. Samples were fired in air at 900, 1300, and 1350 °C for 2, 24, and 12 h, respectively, at 10 °C/min. Prior to the second and third heating steps, samples were reground dry, and the powder subjected to x-ray diffraction (XRD) to monitor phase progression. The powders were pressed again after XRD measurement and before the subsequent heating step. After the final heating step, the target was cooled at 5 °C/min and annealed at 500 °C for 8 h to improve oxygen stoichiometry.[7]

S2.2 Dictionary Indexing Details

Bulk lattice parameters were used in the construction of the DI master patterns. The cubic (c) 3C BSMO lattice parameter was estimated from single crystal data for $Ba_xSr_{1-x}MnO_3$ ($0 \leq x \leq 0.5$), assuming a pseudocubic (pc) lattice following Vegard's law: a_{pc} = 3.883 Å. The hexagonal (h) 4H BSMO lattice parameters were taken from experimental data by Abbas et al.:[8] a = 5.560 Å and c = 9.140 Å. The lattice parameters for GSO were: a= 5.745 Å, b = 7.935 Å, and c = 5.486 Å. Since orientation variations in the films are less than 5° from the 3C $(001)_c$ or 4H $(034)_h$, phase maps are not shown for the films on DSO $(101)_o$; the color between phases and their respective orientations are distinguishable from each other.

S3 X-Ray Reflectivity Measurements

As described in the main text, x-ray reflectivity (XRR) scans were carried out on select $Ba_{0.6}Sr_{0.4}MnO_3$ films to determine their thickness and to estimate growth rate. Since growth was extremely rough on alumina-polished orthorhombic (o) $DyScO_3$ $(101)_o$ substrates, film thicknesses were estimated from the growth rate determined by deposition on colloidal-silica polished substrates. The growth rate was determined by depositing 1000 pulses of BSMO using regular (r)PLD and standard conditions. An experimental XRR scan is shown as a blue line in Fig. S1(a). Simulations using differences in electron density only (not crystal structures because amplitudes of oscillations do not depend on periodicity of the sample) were carried out using the X'Pert Reflectivity software and best fit values are plotted as a red solid line. For this scan, the simulated curve noticeably differs from the experimental curve. The difference can be attributed to interface roughness or density differences, which affects the amplitude [9, 10]. Further refinement of the XRR scan was not pursued. The simulated thickness was 12 nm, resulting in a growth rate of 0.12 Å/pulse.

XRR scans in Fig. S1(b) and (c) correspond to the films from Fig. 4(a) and (b), respectively, deposited under standard conditions with 1000 pulses using rPLD and interval (i)PLD, respectively. Both films were expected to be 12 nm based off of the growth rate determined from Fig. S1(a). The simulated thicknesses values for Figs. S1(b) and (c) were 11 and 15 nm, respectively. Simulated curves fit the measured scans well, indicating that both films had relatively uniform densities and smooth interfaces. Since Figs. S1(a) and (b) were deposited under the same conditions, slight differences in their XRR scans (and thickness values of 12 and 11 nm, respectively) could arise from uncertainties in the fitting parameters of XRR, as well as slight differences of background pressures, atmospheric humidity, predeposition substrate annealing time, or other factors that vary between depositions. These values were acceptable for the purposes of this work.

The XRR scan for the iPLD deposited coherent, single phase film of $Ba_{0.6}Sr_{0.4}MnO_3$ on $DyScO_3$ $(101)_o$ is given in Fig. S1(d) as a blue line. The simulated curve with a 41 nm thickness is given as a red line. The expected thickness was 40 nm, which is close to the targeted value (see main text for further discussion). The dampened amplitude of the oscillations in the 1.4-1.7° 2Θ region is likely from interface roughness effects between layers which were not explored further (this is distinct from large island effects described in the main text).

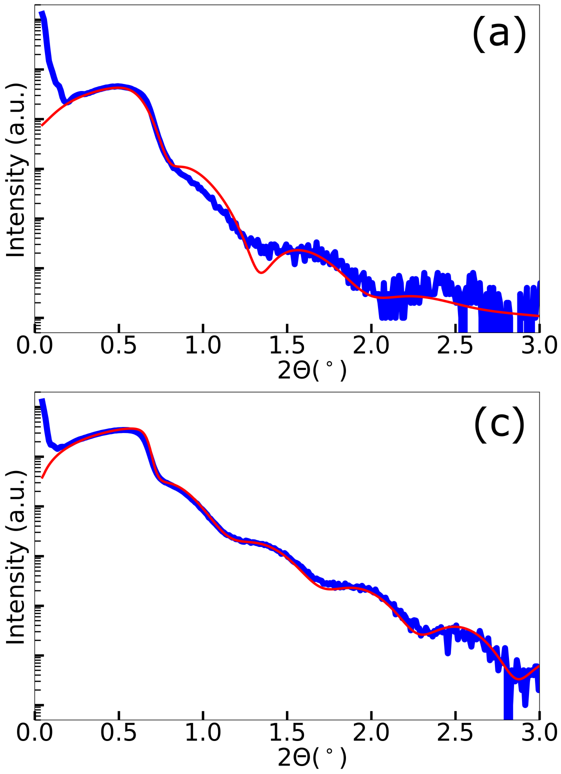

Figure S1: XRR scans (blue solid lines) and simulations (red solid lines) based off differences in electron densities of the film and substrate for: a) a 12 nm rPLD film; b) an 11 nm rPLD film; c) a 15 nm iPLD film; and d) a 41 nm iPLD film. All films were deposited under standard conditions.

S4 Simulated X-ray Diffraction Patterns

The purpose of this section is to demonstrate why it may be possible to detect the 4H (023) and/or (034) film peak in XRD and why we do not find evidence of either peak. The (023) orientation was identified in XRD patterns for a 4H $BaRuO_3$ film on $SrTiO_3$ (100) by Lee et al.[11]. The (034) orientation was identified from EBSD orientation maps of $SrMnO_3$ on a near (100) $SrTiO_3$ grain in a polycrystalline substrate. The rotation of 4H (034) BSMO from the eutactic (001) plane is 54.9°, which is only 0.2° away from the exact rotation need to go from the 3C $SrTiO_3$ eutactic (111) plane to (100) (54.7°). This rotation away from the eutactic plane is driven by the alignment of nearly close-packed planes and directions between film and substrate, observed for $SrMnO_3$ on $SrTiO_3$.[12] For the 4H (023), the rotation away from the eutactic (001) plane is 51.7°, which is 3° away from the ideal rotation. It is therefore expected that the 4H film on $(101)_o$ $DyScO_3$ is oriented as (034). However, depending on the orientation spread in the film, the (023) is also possible to detect using XRD.

XRD patterns for a 4H (034) and 3C (100) oriented sample were simulated using CrystalDiffract[13] and results are shown in Fig. S2 using different alignments. Substrate peaks are not shown here, but are always located to the left of the 3C film peaks. An alignment of 1 assumes a fully-ordered sample with no orientation deviation and 0 assumes a randomly oriented sample. In between the two, there is a statistical distribution of orientations, which can change the intensities of the peaks and give rise to peaks outside of a family of planes.[13] For completely aligned 4H samples in Fig. S2(a), only the 4H (034) peak is seen for a 4H (034)-oriented sample. Compared to the 3C (300) peak, the intensities are approximately the same. The out-of-plane 4H (034) peak is located at $2\Theta = 71.81°$, which is only 0.16° away from the $(303)_o$ DSO peak and is difficult to resolve even with high resolution XRD. When the alignment is decreased to 0.9 for both phases, the (023) becomes visible at $2\Theta =$

48.13° with a slightly higher intensity. It should be noted that in reality, the alignment is not necessarily the same for both phases, so their relative intensities, which are also related to their phase fractions, may vary from what is simulated. Additional factors such as strain in the film or XRD instrument settings can broaden peaks or introduce background noise, furthering the difficulty in detecting $4H$ peaks.

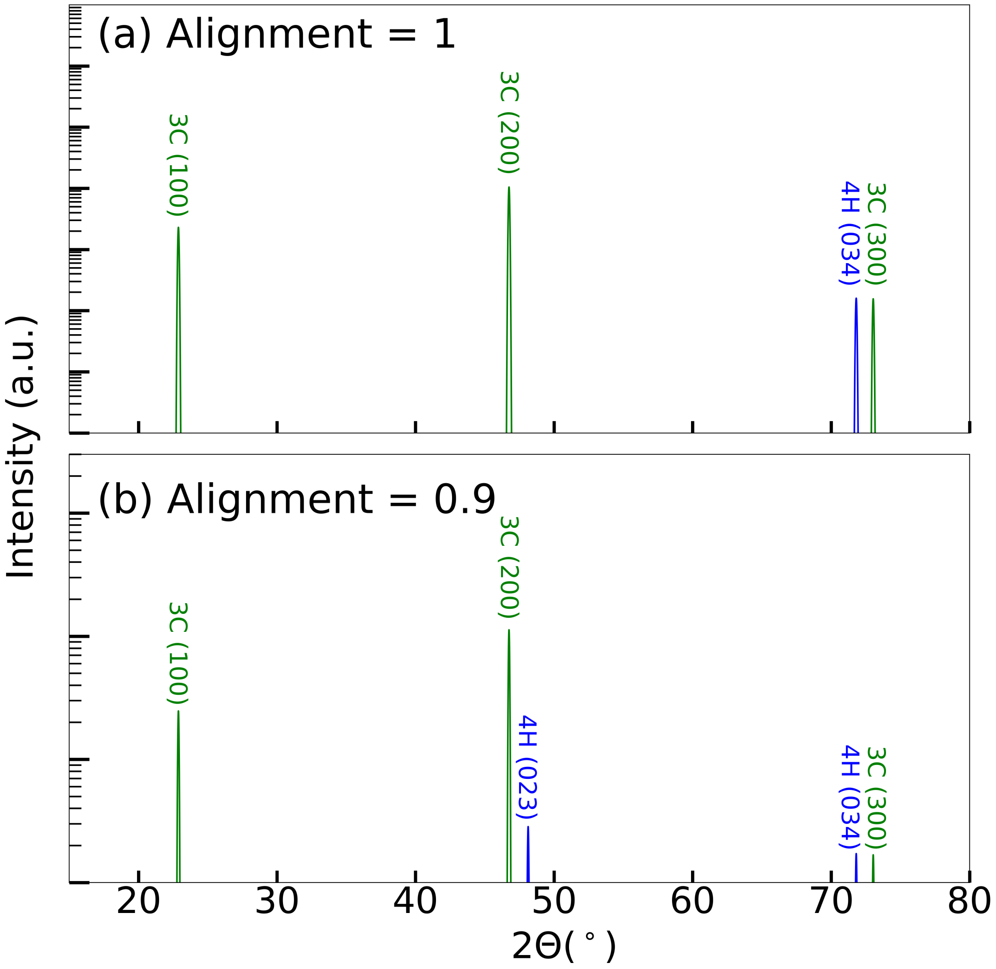

Figure S2: Simulated XRD patterns for the expected $4H$ and $3C$ film peaks on $(101)_o$ DSO (not shown) using alignment values of (a) 1 and (b) 0.9. Green peaks represent the position of $3C$ peaks and blue peaks represent the position of $4H$ peaks.

S5 XRD Comparisons using Lens Optic

XRD patterns reported in the main text were collected using high-resolution (hi-res) optics. In the hi-res optics mode, only signal from the Kα_1 is captured and there is a significant decrease in beam divergence, but these come at the cost of a decrease in total intensity. Here, XRD patterns registered using lower resolution (lens) optics that have a higher intensity (but also have Kα_2 and wider divergence of the beam) are presented, as these allow for observation of lower intensity peaks.

Figure S3 shows the XRD patterns (collected using the lens optics) of the rPLD and iPLD films. The rPLD patterns in Fig. S3(a) and (b) are reproduced from the main text, Fig. 1. The iPLD films in Figs. S3(c)-(e) are the same as in the main text, whose XRD patterns collected with hi-res optics are shown in Figs. 5(a)-(c), respectively. The red (black) vertical lines in these images are at the same angular location for the films (substrates) as those in Figs. 5(a)-(c). The iPLD film peak in Fig. S3(c) is very close to the substrate peak and poorly resolved; it is more clear using the hi-res optics (including thickness fringes). On the contrary, the films' peak locations (maximum values) are more clearly observed for the other two iPLD films (Fig. S3(b) and (c)) using the lens optics, because the films have an inherently lower crystalline quality (more variable strains and possibly overall volume), which cause the hi-res scans to have lower intensity and wider peak widths.

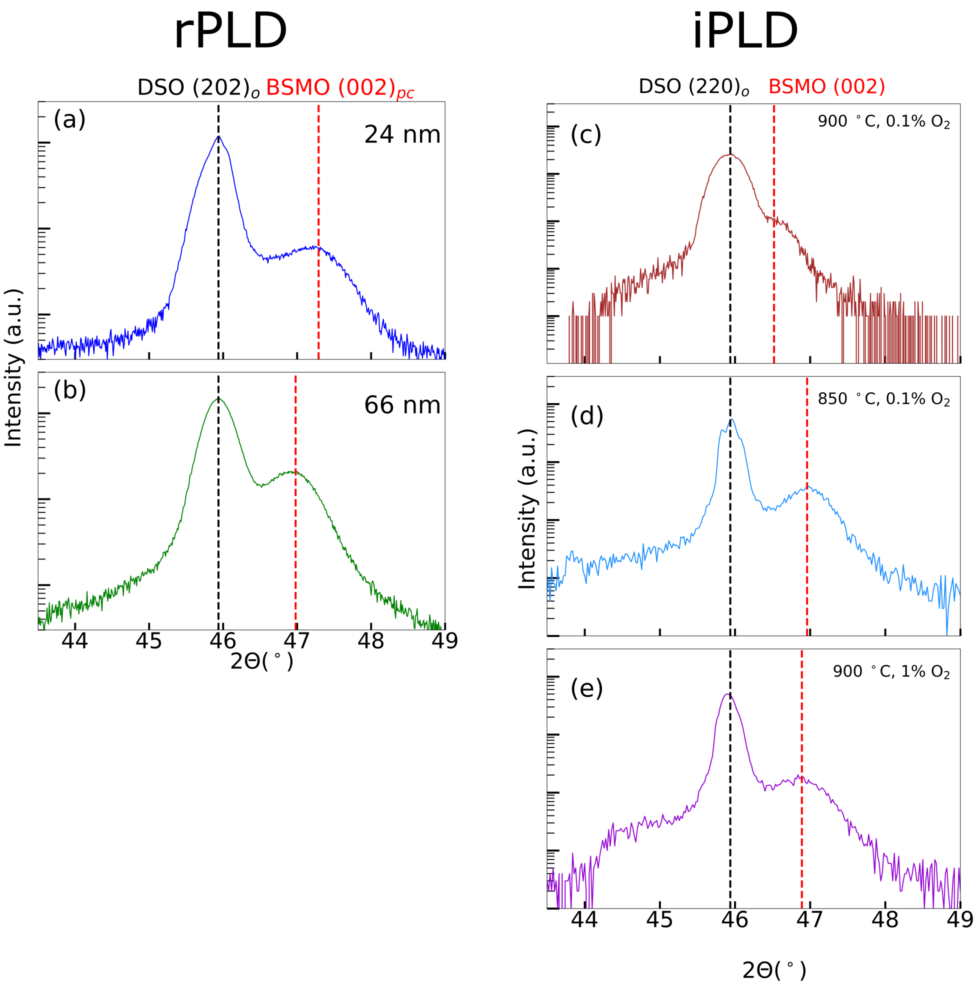

Figure S3: XRD patterns using lens optic from rPLD and iPLD films. (a) and (b) are the 24 and 66 nm films deposited under standard conditions using rPLD. XRD patterns from iPLD films are deposited under (c) standard conditions, (d) $T_s = 850$ °C, and (e) $P = 2$ mTorr 1% O_2 ($P_{O_2} = 2 \times 10^{-5}$ Torr). The red (black) vertical lines are at the same angular location for the films (substrates) in Figs. 5(a)-(c) in the main text.

S6 HAADF-STEM Image of Film Defects

Figure S4(a) shows a HAADF-STEM image of the BSMO film, imaged along the $[110]_{pc}$ zone axis. The growth direction $[001]_c$ points upwards. The brighter area at the bottom of the field is near the substrate, but the interface was not resolved here. The area captured in the image includes several planar defects. The nearly vertical line of contrast on the right side of the image is reminiscent of a low-angle grain boundary (it is marked as such using gb), but may also represent a growth variant boundary (separating symmetrically related variants of different orientations) or an anti-phase boundary (where the crystal units on either side are displaced from one another by a translation). Although the atomic resolution is not very clear near this grain boundary, rows of atoms look misaligned (no one-to-one bonding) going across the boundary (as indicated by the blue lines), indicating that this could be a semi-coherent grain boundary. Additionally, this grain boundary could result from a structural distortion in the substrate, which is then propagated across the interface into the film.[14]

There is also a network of intersecting diagonal boundaries angled approximately 55° from the horizontal rows and marked as twin boundaries (tb) in Fig. S4(a) because these coherent faults are similar to those observed for BSMO films with $x \leq 0.5$ by Langenberg et al.[7] This angle is also the angle formed between $(001)_c$ and $(111)_c$ in the 3C phase, and thus may represent stacking faults (incorrectly stacked eutactic $(111)_c$ planes in the $3C$ stacking sequence) along the $\langle 111 \rangle$. Such stacking faults lead to local hexagonal stacking and some face-sharing of octahedra and can be twin boundaries in cubic structures. Crystal structure alignment of coherently twinned $3C$ unit cells around the $\{111\}$ planar boundaries are shown in Fig. S4(b). In between the boundaries is the misoriented twin domain, which is only one unit cell wide. Importantly, no evidence of extended $4H$ regions was found when taking FFTs of regions within each domain. An example of a such a region is highlighted by the

green box in Fig. S4(a) and enlarged in (c). The FFT of the highlighted region is shown as an inset in (c). All FFTs were similar to the one in Fig. S4(c), which are also similar to the FFT from Fig. 8b). Thus, only local regions of hexagonal stacking may be present, confined to the twin boundary (or stacking fault). Note that this double set of faults also leads to a slight misalignment of the horizontal atom rows on either side of the coherent boundary in Fig.S4(b), which would normally be called an anti-phase boundary. The grain boundary discussed above may arise from the impingement of nuclei having different alignment of such domains emanating from stacking faults.

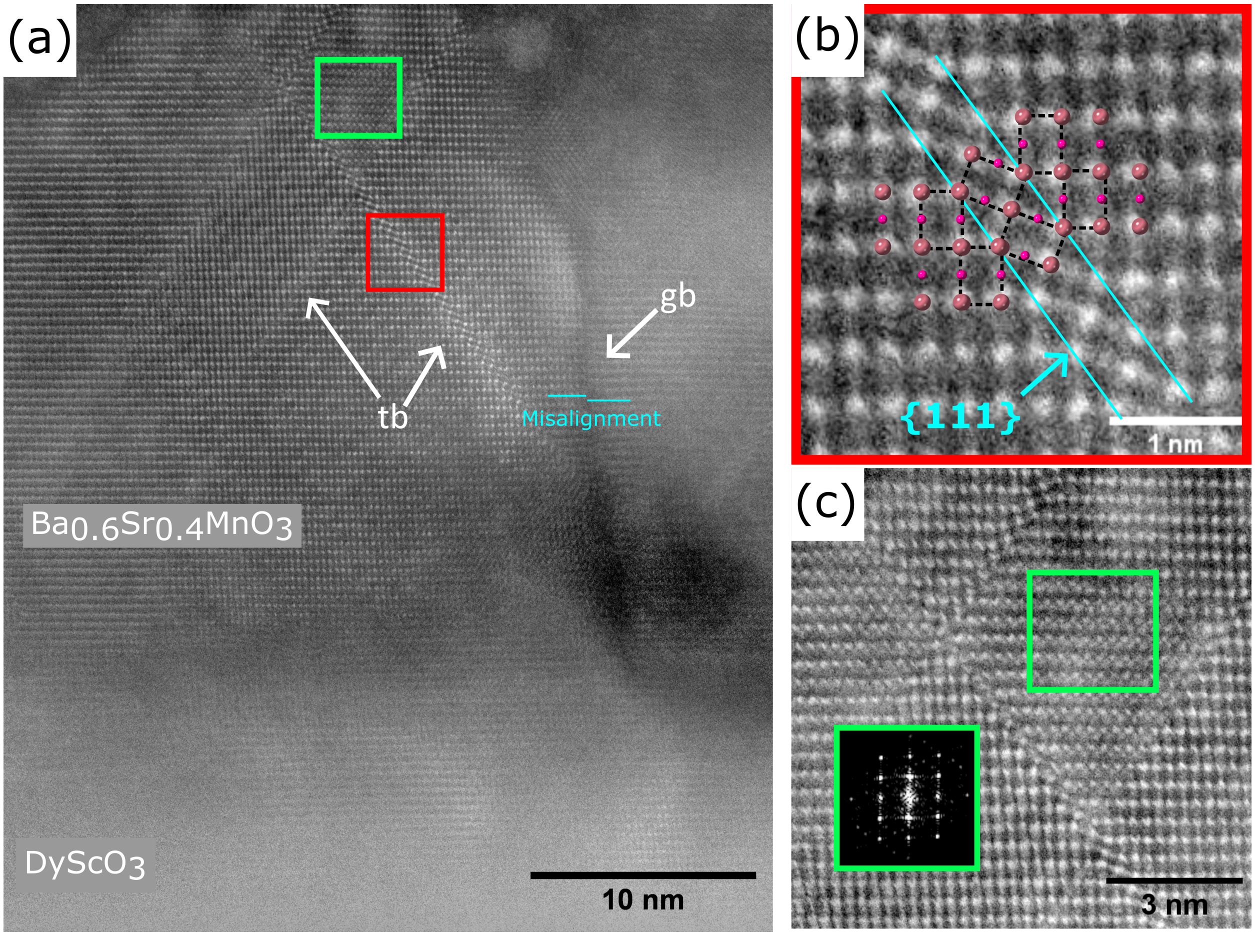

Figure S4: HAADF-STEM image of the [110] cross section of the 40 nm iPLD film. (a) is a large field of view containing the substrate $DyScO_3$ (bottom) and the BSMO film (top). Two twin boundaries are marked as tb (others are visible) and another boundary is marked as gb and described in the text. (b) A closer view of the region in the red box of (a) highlighting the twin boundary atomic structure. The (111) twin plane is denoted by diagonal lines and the Ba/Sr and Mn cations are denoted by large and small spheres. (c) A closer view of the region around the green box in (a) with an FFT from the region within the green box. Domains are separated by twin boundaries.

S7 In-plane Epitaxy and Misorientations in the 40 nm iPLD $Ba_{0.6}Sr_{0.4}MnO_3$ Film

Figure S5 shows inverse pole figure (IPF) maps (from EBSD data) of the film in the X, Y, and Z-directions for an 40 nm iPLD BSMO ($x = 0.6$) film on DSO $(101)_o$. These laboratory directions are aligned close to the pseudocubic <100> axes of DSO $(101)_o$, with Z corresponding to the growth direction. All maps of the film are colored red, with only slight hue variations, indicating that there exists only one orientation in each direction of the film. These orientations, which are near the (100), (010), and (001), indicate that local epitaxial growth exists in all three directions, everywhere across this film.

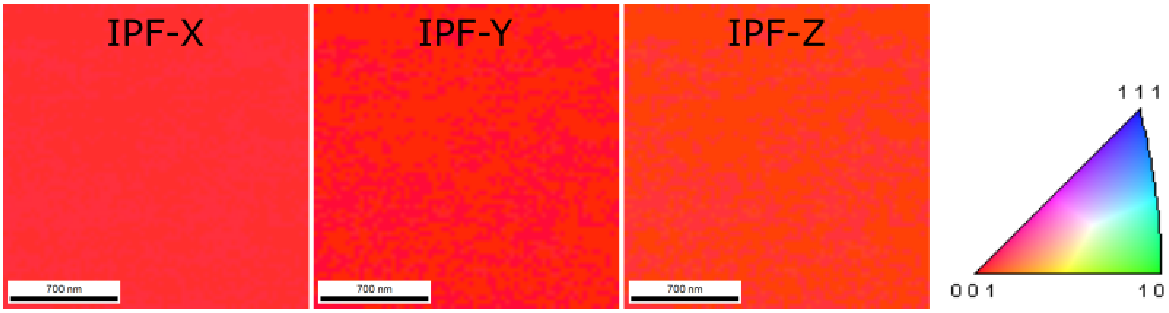

Figure S5: Inverse pole figure (IPF) orientation maps of the 40 nm iPLD BSMO ($x = 0.6$) film deposited on DSO $(101)_o$ under standard conditions. From left to right, maps colors correspond to the plane normal that is parallel in the X, Y, and Z-direction (and Z is in the direction of growth). The IPF color key is shown to the right of the orientation maps. Step sizes and scale bars are 30 nm and 700 nm, respectively.

Figure S6(a) shows the kernel average misorientation (KAM) map of the same 40 nm iPLD BSMO ($x = 0.6$) film. KAM maps show the local average misorientation of a point with all its neighbors (pixels). In the construction of the maps in Fig. S6, only the first nearest neighbors were considered. Local misorientations range from 0° (colored blue) to 2.2° (colored red). A histogram of the values in the KAM map is shown in Figure S6(b). The average misorientation was 0.85°, which is consistent with other epitaxial films analyzed using EBSD.[15–17] Clusters of yellow pixels, outlined by green ones, indicate areas of relatively

higher misorientation between neighboring pixels compared to areas colored blue. These cluster suggest the presence of islands with smoother regions in between islands, which is evident in the AFM image in Fig. 6(a). The difference in local orientation between areas may correlate with relaxations around roughness, in which case the iPLD process could be further optimized, or relaxations from epitaxial strain in some regions, possibly consistent with defects similar to those shown in the prior section (§ S6), or dislocations. Further investigations were not carried out. It is certainly of interest to further explore the impact of such disorder on continued nucleation beyond 40 nm, or whether iPLD can be further optimized to reduce the range of misorientations and extended defects.

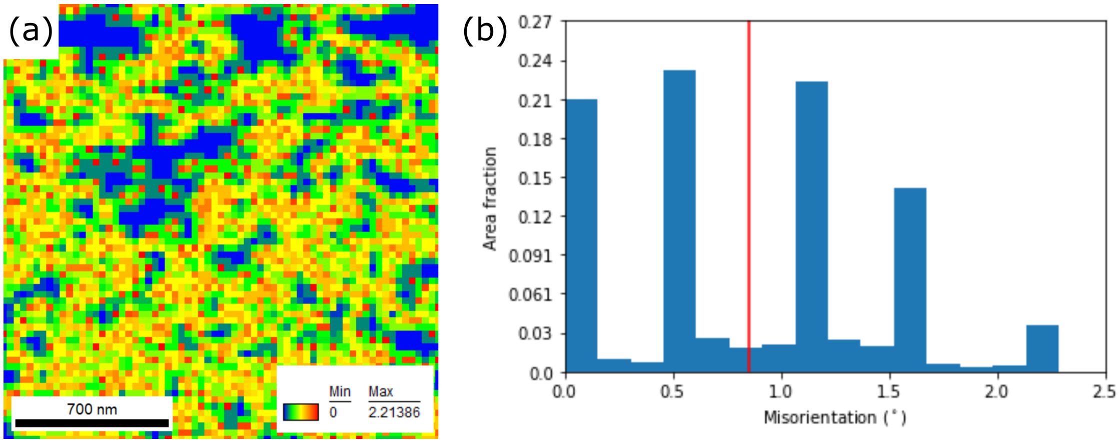

Figure S6: (a) Kernel Average Misorientation map of the 40 nm iPLD BSMO ($x = 0.6$) film deposited on DSO $(101)_o$ under standard conditions. The color scale ranges from blue (0°) to red (2.2°). The step size and scale bar are 30 nm and 700 nm, respectively. A histogram of the misorientation data is shown in (b). The red line indicates the average misorientation.

S8 Misorientations in rPLD and iPLD $Ba_{0.5}Sr_{0.5}MnO_3$

$Ba_{0.5}Sr_{0.5}MnO_3$ (BSMO x=0.5) films were deposited on $DyScO_3$ $(101)_o$ single crystal substrates under standard conditions using rPLD and iPLD methods. The BSMO (x=0.5) target was synthesized similarly to the x=0.6 target, following the protocol by Langenberg et al.[7] Note, this is the only set of experiments for which we used the different composition. Growth rates were estimated from deposition on $SrTiO_3$ (111) under standard conditions but in a 1% oxygen process gas ($P_{O_2} = 2 \times 10^{-5}$ Torr), which we assume approximates that in other mixed gases at the same total pressure. However, growth rates should differ slightly between substrate orientations,[18] and so the estimation of pulse number for one monolayer will be inaccurate. After deposition in standard conditions (as defined in the main text), the actual film thicknesses were measured using XRR to be 15 nm and 20 nm for the rPLD and iPLD films, respectively. This difference in thickness is consistent with the difference between BSMO (x=0.6) rPLD and iPLD films, where the growth rate determined from rPLD underestimates the true growth rate (see main text).

Figure S7 shows the AFM images (a,d), EBSD orientation maps (b,e), and kernel average misorientation (KAM) maps (c,f). The top row is from the rPLD film (a,b,c) and bottom row is from the iPLD film (d,e,f). The RMS roughnesses of Figs. S7(a) and (d) were 5.61 nm and 5.32 nm. While the films' roughnesses do not differ significantly, the rPLD film has a higher density of islands compared to the iPLD film, where a lower density of larger islands is observed. EBSD orientation maps for both films are shown in Fig. S7(b) and (e). Both rPLD and iPLD films are indexed as $3C$ at thicknesses larger than the critical thickness achieved by Langenberg et al.,[7] providing a stronger touchstone between the different deposition conditions used in the two works. There is more variation in the red coloring in the rPLD film, indicating a larger orientation spread around the $(001)_c$ orientation. Both of these indicate a higher crystalline quality to the iPLD film.

Kernel average misorientation (KAM) maps were constructed from EBSD data and shown in Figures S7(c) and (f) for the rPLD and iPLD films, respectively. These maps show that the local misorientation in the iPLD film is much lower, near 0° and blue, compared to the rPLD film, which varies from 0 to 2.7° and has colors from blue to red. The clusters that are green in Fig. S7(f) have slightly slightly higher misorientations and these are suggestive of the large islands (or clusters of islands) seen in the AFM image (d). There is also a green line that goes across the iPLD KAM map in Fig. S7(f), which may represent some defect in the film (such as a planar boundary that intersects the surface). Overall, the KAM maps show that lower local misorientations are found in iPLD compared to rPLD, which is consistent with relaxation processes that can occur during the interval without deposition. This control over crystalline quality is also consistent with lower accumulated defects and more persistent $3C$ nucleation, as discussed in the main text for BSMO (x=0.6).

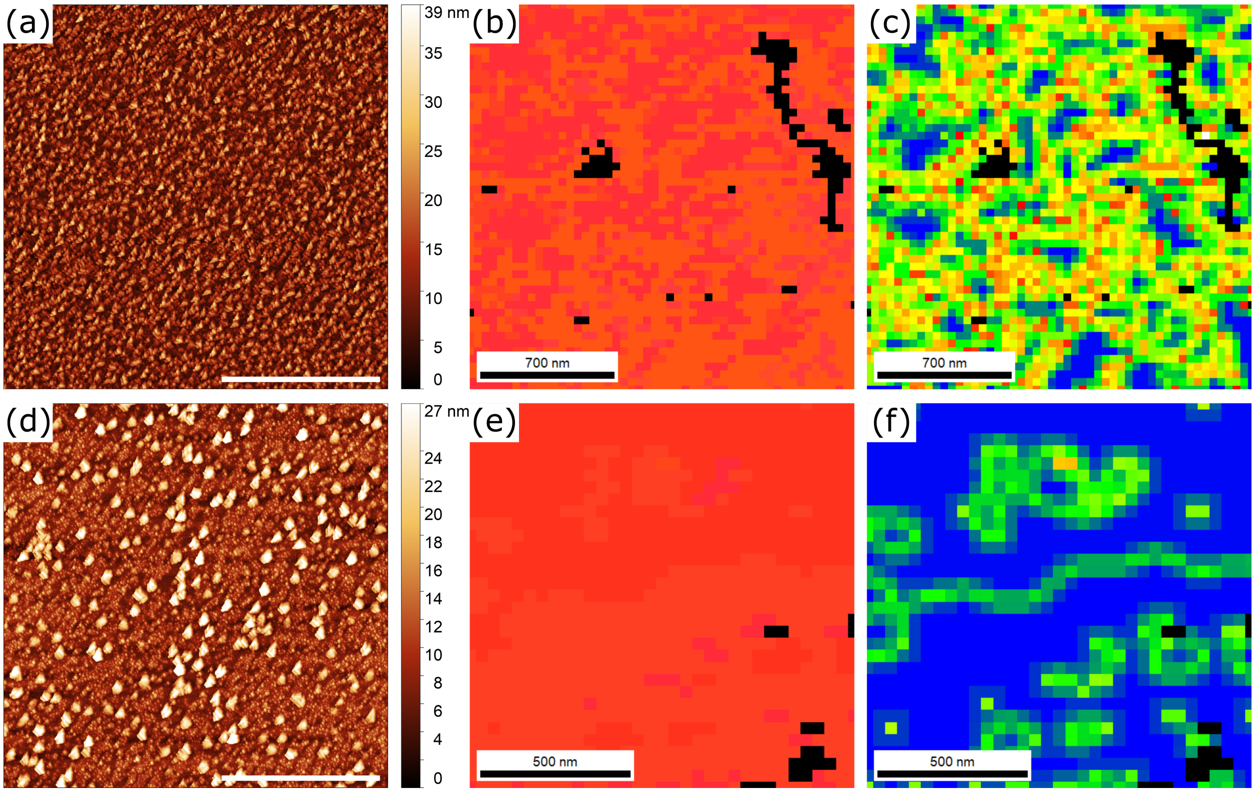

Figure S7: AFM images (a,d), EBSD orientation maps (b,e), and kernel average misorientation (KAM) maps (c,f) for BSMO (x=0.5) films on $DyScO_3$ $(101)_o$. The top row (a,b,c) are from a 15 nm rPLD film and the bottom row (d,e,f) are from a 20 nm iPLD film. Scale bars in the AFM images are 4 μm. The color key for orientation maps (b,e) is the cubic key shown in Fig. 2 of the main text: the $3C$ orientations for these films are distributed close to $(001)_c$. The color scale in the KAM maps (c,f) range from from blue (0°) to red (2.7°).

References

(1) Søndenå, R.; Stølen, S.; Ravindran, P.; Grande, T.; Allan, N. L. Corner- versus Face-Sharing Octahedra in $AMnO_3$ Perovskites (A = Ca, Sr, and Ba). *Phys. Rev. B* **2007**, *75*, 184105.

(2) Goldschmidt, V. M. Die Gesetze der Krystallochemie. *Naturwissenschaften* **1926**, *14*, 477–485.

(3) Marthinsen, A.; Faber, C.; Aschauer, U.; Spaldin, N. A.; Selbach, S. M. Coupling and Competition between Ferroelectricity, Magnetism, Strain, and Oxygen Vacancies in $AMnO_3$ Perovskites. *MRS Commun.* **2016**, *6*, 182–191.

(4) Negas, T.; Roth, R. S. Phase Equilibria and Structural Relations in the System $BaMnO_{3-x}$. *J. Solid State Chem.* **1971**, *3*, 323–339.

(5) Dabrowski, B.; Chmaissem, O.; Mais, J.; Kolesnik, S.; Jorgensen, J. D.; Short, S. Tolerance Factor Rules for $Sr_{1-x-y}Ca_xBa_yMnO_3$ Perovskites. *J. Solid State Chem.* **2003**, *170*, 154–164.

(6) Kuroda, K.; Shinozaki, K.; Uematsu, K.; Mizutani, N.; Kato, M. Phase Relation and Oxygen Deficiency of $SrMnO_{3-x}$. *Nippon Kagaku Kaishi* **1977**, *1977*, 1620–1625.

(7) Langenberg, E.; Guzmán, R.; Maurel, L.; Martínez de Baños, L.; Morellón, L.; Ibarra, M. R.; Herrero-Martín, J.; Blasco, J.; Magén, C.; Algarabel, P. A.; Pardo, J. A. Epitaxial Stabilization of the Perovskite Phase in $(Sr_{1-x}Ba_x)MnO_3$ Thin Films. *ACS Appl. Mater. Interfaces* **2015**, *7*, 23967–23977.

(8) Abbas, S. K.; Aslam, M. A.; Amir, M.; Atiq, S.; Ahmed, Z.; Siddiqi, S. A.; Naseem, S. Electrical Impedance Functionality and Spin Orientation Transformation of Nanostructured Sr-Substituted $BaMnO_3$ Hexagonal Perovskites. *J. Alloys Compd.* **2017**, *712*, 720–731.

(9) Chason, E.; Mayer, T. M. Thin Film and Surface Characterization by Specular X-ray Reflectivity. *Crit. Rev. Solid State Mater. Sci.* **1997**, *22*, 1–67.

(10) Yasaka, M. X-ray Thin-Film Measurement Techniques. *Rigaku J.* **2010**, *26*.

(11) Lee, M. K.; Eom, C. B.; Lettieri, J.; Scrymgeour, I. W.; Schlom, D. G.; Tian, W.; Pan, X. Q.; Ryan, P. A.; Tsui, F. Epitaxial Thin Films of Hexagonal $BaRuO_3$ on (001) $SrTiO_3$. *Appl. Phys. Lett.* **2001**, *78*, 329–331.

(12) Zhou, C.; De Graef, M.; Dabrowski, B.; Rohrer, G. S.; Salvador, P. A. Combinatorial Substrate Epitaxy Investigation of Polytypic Growth of $AEMnO_3$ (AE= Ca, Sr). *J. Am. Ceram. Soc.* **2020**, *103*, 2225–2234.

(13) CrystalMaker Softward Ltd, Oxford, England.

(14) Sharma, P.; Huang, Z.; Li, M.; Li, C.; Hu, S.; Lee, H.; Lee, J. W.; Eom, C. B.; Pennycook, S. J.; Seidel, J.; Ariando; Gruverman, A. Oxygen Stoichiometry Effect on Polar Properties of $LaAlO_3/SrTiO_3$. *Adv. Funct. Mater.* **2018**, *28*, 1707159.

(15) Zhang, Y.; Koch, C. C.; Ma, S. G.; Zhang, H.; Pan, Y. Fabrication Routes. *High-Entropy Alloy. Fundam. Appl.* **2016**, 151–179.

(16) Win, T. Z.; Inaba, K.; Kobayashi, S.; Kanetake, Y.; Nakamura, Y. Epitaxy of Tin Dioxide on Titanium Dioxide by Mist Chemical Vapor Deposition. *Trans. Mater. Res. Soc. Japan* **2020**, *45*, 173–178.

(17) Scheerbaum, N.; Lai, Y. W.; Leisegang, T.; Thomas, M.; Liu, J.; Khlopkov, K.; Mc-Cord, J.; Fähler, S.; Träger, R.; Meyer, D. C.; Schultz, L.; Gutfleisch, O. Constraint-Dependent Twin Variant Distribution in Ni_2MnGa Single Crystal, Polycrystals and Thin Film: An EBSD Study. *Acta Mater.* **2010**, *58*, 4629–4638.

(18) Woo, S.; Choi, H.; Kang, S.; Lee, J.; David, A.; Prellier, W.; Kim, Y.; Kim, H. Y.; Choi, W. S. Surface-Orientation-Dependent Growth of $SrRuO_3$ Epitaxial Thin Films. *Appl. Surf. Sci.* **2020**, *499*, 143924.
